# The elements of life and medicines

**DOI:** 10.1098/rsta.2014.0182

**Published:** 2015-03-13

**Authors:** Prinessa Chellan, Peter J Sadler

**Affiliations:** Department of Chemistry, University of Warwick, Coventry CV4 7AL, UK

**Keywords:** periodic table, essential elements, genetic codes, inorganic chemistry, coordination chemistry, metals in medicine

## Abstract

Which elements are essential for human life? Here we make an element-by-element journey through the periodic table and attempt to assess whether elements are essential or not, and if they are, whether there is a relevant code for them in the human genome. There are many difficulties such as the human biochemistry of several so-called essential elements is not well understood, and it is not clear how we should classify elements that are involved in the destruction of invading microorganisms, or elements which are essential for microorganisms with which we live in symbiosis. In general, genes do not code for the elements themselves, but for specific chemical species, i.e. for the element, its oxidation state, type and number of coordinated ligands, and the coordination geometry. Today, the biological periodic table is in a position somewhat similar to Mendeleev's chemical periodic table of 1869: there are gaps and we need to do more research to fill them. The periodic table also offers potential for novel therapeutic and diagnostic agents, based on not only essential elements, but also non-essential elements, and on radionuclides. Although the potential for inorganic chemistry in medicine was realized more than 2000 years ago, this area of research is still in its infancy. Future advances in the design of inorganic drugs require more knowledge of their mechanism of action, including target sites and metabolism. Temporal speciation of elements in their biological environments at the atomic level is a major challenge, for which new methods are urgently needed.

## 1. Introduction

The question ‘Which elements are essential for human life?’ is frequently asked and seems simple enough to answer. However, the longer you consider it, the more complicated the answer becomes. We know the sequence of the human genome, and surely all essential elements are coded for—but are they? And if so, how? Moreover, an element can be both good and bad for life. For example, there is approximately 80 mg of Cu in the body, and copper, an essential trace element, plays several roles in human physiology, including the development of connective tissue, bone and nerve coverings, but chronic copper toxicity, while rare, can lead to liver damage, and acute copper intoxication can lead to severe gastrointestinal effects [1].

Our answer will also depend on how we define ‘human life’. We live in symbiosis with microorganisms. Approximately 500–1000 species of bacteria live in the human gut [2] and there are more than 10 times as many bacterial cells as human cells in the body [3,4]. If these bacteria have a different requirement for elements than our own cells, do we also call these elements essential for human life?

The human body contains at least 60 detectable chemical elements, however, only about 25 of these elements are believed to participate in the healthy functioning of the human body [5]. The essentiality of additional elements is still in contention, among them arsenic, chromium, boron and lithium, but most studies have been carried out on laboratory mammals (and may or may not be applicable to humans) and the ultra-trace levels of some elements that are present in the body make it difficult to establish their nutritional value [5,6].

Forty to 50 years ago was a period during which nutritionists were carrying out dietary experiments to determine the essentiality of elements, often using rodents. Such experiments were expensive and time-consuming, with the need to purify all components of the diet and to make sure the animals were living under defined conditions. Nutritional researchers included Schwarz, who is not only credited with discovery of the essentiality of selenium [7–10] but also investigated the essentiality of many other elements including Sn [11], Si [12] and Cd [13]. Some of his studies showed that despite being considered non-essential, cadmium, for example, influenced the growth of rats. The growth of weaning rats fed on diets supplemented by Cd at levels normally found in foods showed a small but consistent increase, e.g. 13% growth increase for 0.2 ppm Cd^2+^ sulfate.

Nielsen has discussed the establishment of a recommended daily allowance (RDA) for elements to safeguard against deficiencies. These include not only proven essential elements but also elements thought to be beneficial but for which essentiality has not been proved [14–17]. It can also be argued that nutritional requirements should include consideration of the total health effects of nutrients, not just their roles in preventing deficiency pathology alone. Ultratrace elements with health benefits which were thought to merit specific RDAs include I, Se, Mn, Mo, Cr and B, as well as Co as vitamin B12. Elements with ‘apparent beneficial intake’ included arsenic, fluorine, lithium, nickel, silicon and vanadium [17–20]. It has also been suggested that a healthful diet should provide an appropriate intake of Al, Br, Cd, Ge, Pb, Rb and Sn [18].

Nutritional research on the essentiality of trace elements does not appear to gain such prominence today. Care has to be taken even when extrapolating the requirements of rodents to humans because there are differences in their genome sequences, and therefore protein sequences. The levels of some low molecular weight metabolites (e.g. metal chelating agents such as citrate) may also change.

This paper is not intended to be a comprehensive review, but attempts to highlight current knowledge of essential elements and elements useful in diagnosis and therapy and notable features in their chemistry which relate to their biological activity. We shall find that any code for an essential element that we can recognize is nearly always a code for a specific species of that element, often requiring, for a metal ion, recognition of a particular oxidation state, coordination number, geometry and ligand set.

We travel through the periodic table group by group, starting with group 1 (hydrogen+alkali metals), followed by groups 2, 3–12 (the transition metals, lanthanides and actinides), 13–17 (p-block elements, mostly non-metals and metalloids) and finally group 18, the noble gases. We conclude with a list of elements for which there is good evidence of essentiality for humans. Perhaps surprisingly, the list is shorter than is believed. The uncertainties in the list will hopefully stimulate much-needed future research in this field.

## 2. Group 1: hydrogen and the alkali metals

There is no doubt about the essentiality of *hydrogen* (*Z*=1), the most abundant element in our universe. Hydrogen can be placed in either group 1 or 17. The proton H^+^ stands alongside the alkali metal ions, but hydride H^−^ is also important in the body, not as free ion, but as donated by the reduced coenzyme nicotine adenine dinucleotide, NAD(P)H.

The control of pH is important in the body. In most body fluids and tissues, the pH is tightly held at pH 7.4 and it is a sign of distress if there is a significant deviation from this value. The control of pH is achieved by buffering, for example, by carbonic acid (H_2_CO_3_)/bicarbonate in blood and by proteins. In tumour tissues, the pH can drop to 6–7 [21], in lysosomes to 4–5 [22] and in endosomes the pH drops to 5.5 and plays a key role in, for example, the release of Fe^3+^ from its transport protein transferrin [23]. Down the gastrointestinal tract, the pH drops from 6 to 6.5 in the duodenum, to 3.5–7 in the large intestine, to 1–3 in the stomach. The consequences of this for the speciation of some elements are intriguing (e.g. see fluorine).

Although most of natural hydrogen is protium ^1^H (99.9885%), there is a small amount of the heavier isotope deuterium ^2^H (0.0115% abundant) in everything we eat and drink. The consequences of kinetic isotope effects for life and the slowing down of biochemical reactions involving the heavier isotopes are interesting and well recognized in the geochemical world [24]. Organisms can incorporate lighter isotopes of transition metals preferentially, which may have consequences for evolution on a long time scale [25]. The pharmaceutical industry uses ^2^H in multiple ways, including for kinetic isotope effects which can slow down drug metabolism (e.g. a C−D bond is cleaved 6–10× more slowly than a C−H bond) [26], for tracing by mass spectrometry, and in medical imaging [26]. Radioactive tritium (^3^H, half-life 12.3 yr, β^−^ emitter) is used as a radiotracer. As far as we know, hydrogen gas is not used by man. However, H_2_ is an important reductant used by a wide range of bacteria. Hydrogenase enzymes are present in photosynthetic bacteria, nitrogen fixers, cyanobacteria, strict anaerobes, and *Salmonella* and *Escherichia coli* species.

**Figure 1.**
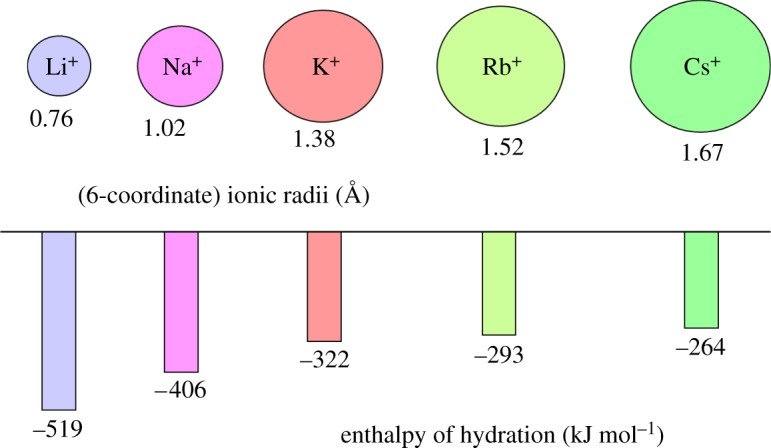
Ionic radii and hydration enthalpies of alkali metal ions [27,28]. These key properties have a major influence on their different biological activities.

*Lithium* (*Z*=3) is not thought to be an essential element, but is present, as Li^+^, in some natural waters, especially ‘spa’ waters (thermal baths) and in some commercial bottled mineral drinking water. There is *ca* 2.4 mg Li in the body.^1^ Perhaps, it has beneficial effects at these low levels. In medicine, lithium salts are widely used for treatment of bipolar disorders (BDs). Li^+^ is a very small ion (6-coordinate radius 0.76 Å, figure 1) with a high hydration enthalpy (−519 kJ mol^−1^).

The symptoms of lithium deficiency in humans are believed to manifest primarily as behavioural abnormalities. A link between low lithium intake and altered behaviour and aggressiveness has been reported [29–31]. As a medicine, lithium is best recognized for its anti-manic properties [32]. It is often administered in the form of lithium carbonate, as a psychiatric drug. More than 2 million American adults, or *ca* 1% of the population 18 years or older, suffer from BD [33]. A recent study was conducted on the influence of lithium on the peripheral blood gene expression profiles of patients with BD [34]. For bipolar patients who responded to lithium, the genes which protect against cell death (including Bcl2 and IRS2) were upregulated, while those which promote cell death were downregulated, including the pro-apoptotic genes known as BAD and BAK1 [34]. These results suggest that increased expression of BCL2 and related genes is necessary for the therapeutic effects of lithium.

Lithium is an inhibitor of the enzyme glycogen synthase kinase-3β (GSK-3β) which is responsible for the hyper-phosphorylation of the tau protein in Alzheimer's disease [33]. A link between genetic variations in the gene encoding glutamate decarboxylase-like protein 1 (*GADL1*) and response to lithium maintenance treatment for bipolar I disorder has been found in patients of Han Chinese descent [35]. The two single-nucleotide polymorphisms (SNPs), rs17026688 and rs17026651, and the *GADL1* variant IVS8 + 48delG are useful markers to predict the response to lithium treatment of patients of Asian descent who have bipolar I disorder.

*Sodium* (*Z*=11) and *potassium* (*Z*=19) are both essential elements that occur at high concentrations in the body (totals of *ca* 112 g and 160 g, respectively). Their crucial roles in cellular homeostasis are well established and they have numerous functions [36]. The biochemistries of Na^+^ and K^+^ are similar although the ions are distinguishable on the basis of their ionic radii (1.02 versus 1.38 Å for 6-coordination) and hydration enthalpies (−406 versus −322 kJ mol^−1^), figure 1. There are specific protein pumps for Na^+^ and K^+^ in cell membranes (Na/K ATPases), which can distinguish between the two ions and maintain plasma Na^+^ at an elevated concentration (140 mM) and intracellular Na^+^ at a lower concentration (12 mM), while the reverse is true for K^+^ (5 versus 140 mM), generating electrical potential gradients responsible for nervous impulses [36]. The process of moving sodium and potassium ions across the cell membrane by the enzyme Na^+^/K^+^-ATPase is an active transport process involving the hydrolysis of adenosine triphosphate (ATP) to provide the necessary energy.

**Figure 2.**
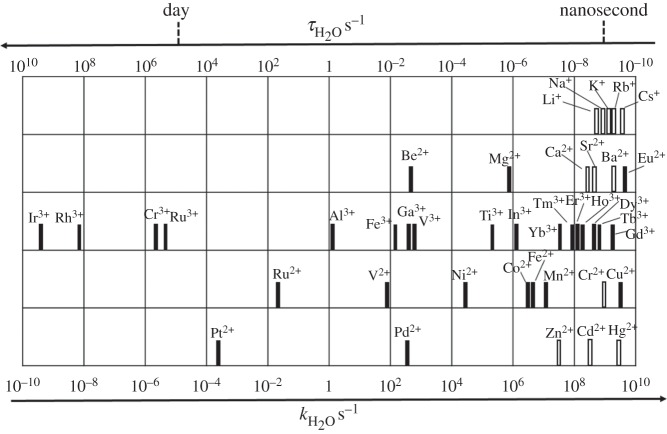
Rates (and lifetimes) of water exchange on metal ions (adapted from [37]). Note the faster exchange on Ca^2+^ compared with Mg^2+^ and the very slow exchange on some transition metal ions. However, the rate of exchange can also show a marked dependence on the other ligands bound to the metal.

Na^+^/K^+^-ATPase controls the transport of three Na^+^ ions to the outside of the cell and the transport of two K^+^ ions to the inside. This unbalanced charge transfer contributes to the separation of charge across the membrane. The sodium–potassium pump is an important contributor to the action potential produced by nerve cells. This pump is called a P-type ion pump because the ATP interactions phosphorylate the transport protein and causes a change in its conformation. For neurons, the Na^+^/K^+^-ATPase can be responsible for up to two-thirds of the cell's energy expenditure. Such transport relies on the kinetic lability of bound water molecules (lifetimes *ca* nanoseconds, figure 2) which are rapidly stripped off as the ions pass into the channel.

Potassium also has a natural radioisotope, ^40^K, with an abundance of 0.012%, a β^−^ emitter with a half-life of *t*_1/2_=1.3×10^9^ yr. There is roughly 0.0169 g of ^40^K present in a typical human body, giving *ca* 266 000 disintegrations per minute, *ca* 4400 disintegrations per second or 4.4 kBq of activity [38].

*Rubidium* (*Z*=37) is present in virtually all animal tissues, but is not thought to be essential. In an average adult (80 kg), there is *ca* 37 mg of Rb. Similar to potassium, it is present in large concentrations in muscle tissue, red blood cells and viscera, and is also similar in its biochemistry [39]. Rubidium-82 which decays by electron capture is an attractive radionuclide for positron emission tomography (PET). It can be generated without a cyclotron and has fast serial rest/stress imaging as a consequence of its short half-life (75 s) [40]. It is used clinically for PET myocardial perfusion imaging (MPI) [41]. A protocol has been developed for simultaneous measurement of myocardial blood flow (MBF) and MPI using ^82^Rb high-count-rate PET [40]. This procedure shows low selectivity for individual patient anatomy or injection site making it a promising tool for obtaining accurate MBF data without loss of image quality [40].

*Cesium* (*Z*=55) is a non-essential, trace element in the body. About 1.6 mg of Cs is distributed in muscle, bone and blood [42]. ^131^Cs (electron capture decay, half-life 9.7 days) has found application in oncology as a treatment for prostate cancer. It is used in brachytherapy, where radioactive seeds are implanted in or near the tumour, exposing it to a high dose of radiation while minimizing radiation exposure of healthy tissues (http://www.isoray.com/). A central nervous system (CNS)-active carborane containing cesium has recently been reported to have an antidepressant effect on mice [43] by inhibiting pore formation by the cation-selective purinergic receptor P2X_7_R ion channel.

## 3. Group 2: the alkaline earths

*Beryllium* (*Z*=4) is not an essential element (only *ca* 3 μg in the body) and is best known for the high toxicity of many of its compounds—especially for its sensitivity and allergic-type responses. Metal–peptide coordination chemistry appears to play a key role in such immunogenic responses. Be^2+^ is a very small ion (4-coordinate ionic radius 0.41 Å) and consequently highly acidic in water, with a p*K*_a_ of *ca* 3.5 for [Be(H_2_O)_4_]^2+^ and a tendency to form polymeric hydroxide-bridged species.

Allergic attacks are launched by the human immune system when the body is exposed to certain metal ions through skin contacts, inhalation and metal-containing artificial body implants [44]. The magnitude of these allergic reactions can range from simple annoyances to life-threatening systemic illness. Currently, the most widely studied human metal hypersensitivities are to nickel and beryllium. αβ T cells play a crucial role in these hypersensitivity reactions. Metal ions (plus their ligands) work as haptens, small molecules that can stimulate an immune response only when attached to a large carrier such as a protein, binding to the surface of the major histocompatibility complex (MHC) [45]. This results in a modification of the binding surface of MHC and activates the immune response of T cells. Metal-specific αβ T-cell receptors (TCRs) are usually MHC restricted (i.e. T cells recognize the peptide antigen only when it is bound to the body's own MHC molecule), especially MHC class II (MHCII) restricted.

**Figure 3.**
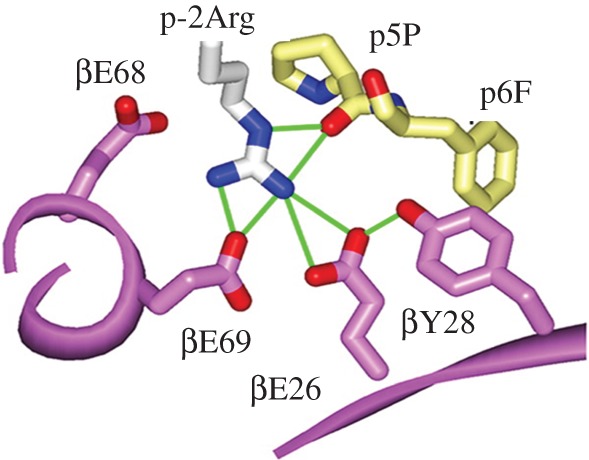
The three glutamate side-chains in the peptide binding groove of the cell-surface receptor protein HLA-DP2 which may form the key Be^2+^ binding site (adapted from [48]).

Despite numerous models being proposed, the mechanisms and molecular basis of metal hypersensitivity are still poorly understood. Chronic beryllium disease (CBD) is a hypersensitivity disorder triggered by beryllium exposure in the workplace. CBD is characterized by the build-up of beryllium-specific CD4^+^ T cells in the lung and granulomatous inflammation [46]. Depending on genetic susceptibility and the nature of the exposure, this can occur in 2–16% of exposed workers. This susceptibility has been linked to HLA-DP alleles containing a glutamic acid at the 69th position (Glu^69^) of the β-chain [47]. The HLA-DP alleles can present Be to T cells equivalent to those associated with genetic susceptibility, suggesting that the HLA contribution to CBD is based on the ability of those molecules to bind and present Be to T cells [48] (figure 3).

The precise Be species needed for T-cell activation is unidentified and it is possible that T cells may not actually distinguish the bound Be complex. The mimotopes and endogenous self-peptides that bind to MHCII and Be, forming a complex recognized by pathogenic CD4^+^ T cells in CBD have been identified [49]. Aspartic and glutamic acid residues at p4 and p7 in these peptides surround the putative Be-binding site and work with HLA-DP2 in Be coordination. In addition to the function of conventional MHC-bound peptides in attaching to MHC and interacting with TCR, it seems clear that there is a novel function for the peptides in metal hypersensitivity, that of metal ion capture. This progression can lead to the transformation of a self-peptide into a neoantigen.

*Magnesium* (*Z*=12) is the fourth most abundant element in the human body, a total of *ca* 25 g. This is distributed in bones (60%), skeletal muscles and soft tissues (30–40%) and extracellular fluids (1%). Mg^2+^ is an essential cofactor in the mechanisms of replication, transcription and translation of genomic information [50–53]. It is also involved in stabilization of lipid membranes, nucleic acids and ribosomes [50,52–54] and plays a crucial role in metabolic networks and signalling cascades, where it participates in regulating enzyme activity and directing macromolecules to particular complexes or cellular locations [55].

Despite magnesium's impact on human health, little is known about the molecular mechanisms that regulate magnesium transport and storage. MgtE and CorA are prokaryotic Mg^2+^-transport systems and are distinctive among membrane proteins containing a unique arrangement of ten transmembrane-spanning α-helices [55]. Both MgtE and CorA sense and respond to physiologically relevant, intracellular Mg^2+^ levels through dedicated regulatory domains. Within these domains, multiple primary and secondary Mg^2+^-binding sites serve to staple these ion channels into their respective closed conformations, implying that Mg^2+^ transport is well guarded and very tightly regulated.

Human MAGT1, magnesium transporter 1, is a critical regulator of the minimum level of intracellular free Mg^2+^ ions. A genetic deficiency in MAGT1 can result in a predisposition to lymphoma as well as elevated levels of Epstein–Barr virus (EBV) [56]. Decreased levels of intracellular free Mg^2+^ have been linked to defective expression of the natural killer activating receptor NKG2D in both natural killer (NK) and CD8^+^ T cells as well as impairment of cytolytic responses to EBV [57]. The intracellular Mg^2+^ and NKG2D levels are restored when MAGT1-deficient patients are given magnesium supplements, and a reduction in EBV-infected cells *in vivo* is also observed [57]. These results show a link between NKG2D cytolytic activity and EBV antiviral immunity in humans.

The biochemistries of Ca^2+^ and Mg^2+^ are similar, although Mg^2+^ is the smaller ion (radius 0.72 versus 1.00 Åfor 6-coordination) and exchanges its bound water ligands more slowly (half-life 10^−5^ versus 10^−8^ s), with Mg^2+^ dominating inside cells (15–20 mM) [6].

Despite the high level of *calcium* (*Z*=20) in the body (*ca* 1.1 kg), and the high total concentration in cells (1–10 mM), free Ca^2+^ at nanomolar and micromolar concentrations regulates skeletal and cardiac muscle contraction by inducing structural changes in the Ca^2+^-sensor protein troponin, a gene product [58]. Control over Ca^2+^ coordination by proteins and small ligands ensures that blood plasma can remain supersaturated in Ca^2+^ without continuously depositing calcium phosphate mineral in bone. Ca^2+^ ATPases in the plasma membrane export Ca^2+^ from all eukaryotic cells and in man are the products of four separate genes [59].

In bones and teeth, the creation and mineralization of extracellular collagen matrix is controlled by osteoblasts and odontoblasts. By contrast, osteoclasts remove bone mineral and bone matrix, making bone cells important for regulation of the formation and resorption of bone. This is a key step in regulating body Ca^2+^ as well as Mg^2+^ and phosphate. Osteoclasts can lower the pH to as low as 4.5 inside the bone-resorbing compartment due to the action of their plasma membrane H^+^ pump, thus assisting bone mineral mobilization [60] while simultaneously secreting lysosomal enzymes such as acid phosphatase, cathepsin K, matrix metalloproteinase 9, and gelatinase [61] that digest the organic matrix. Vast amounts of Ca^2+^ are absorbed by mature osteoclasts during resorption and their survival is ensured by Na^+^/Ca^2+^ exchangers that prevent harmful Ca^2+^ overload in the cytoplasm by extruding Ca^2+^ ions into extracellular space [62,63].

Although there is about 0.4 g of *strontium* (*Z*=38) in the body, it is not thought to be essential. It is handled in the body in a similar manner to calcium. Some of its effects appear to be beneficial. Strontium salts are well known toothpaste additives. Regular tooth brushing with a strontium-supplemented toothpaste increases the Sr content in the exposed enamel, which can be an advantage in the prevention of cariogenesis and perhaps strengthens the enamel [64].

**Figure 4.**
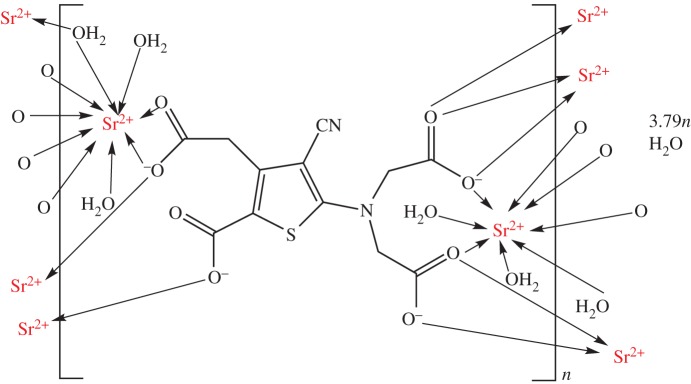
The polymeric structure of strontium ranelate. Each monomer unit contains two Sr^2+^ ions.

The drug strontium(II) ranelate [65] aids bone growth, increases bone density and decreases the incidence of fractures in osteoporotic patients [66]. X-ray diffraction analysis of the drug [Sr_2_(ranelate)] reveals a polymeric structure with each Sr^2+^ ion bound to carboxylate and water O atoms (figure 4) [67]. During clinical trials, women on the drug displayed a significant 12.7% increase in bone density compared to the placebo group (1.6% decrease). Half of this increase in bone density was believed to be a consequence of the higher atomic weight of Sr compared with calcium and the other half was a real increase in bone mass [34,68]. Doses of up to 2 g d^−1^ have been administered [69]. Use of strontium salt additives in topical formulations reduces the signs and symptoms of irritant contact dermatitis [70]. This type of allergy affects many people who use topical drugs, cosmetics and personal care products.

Strontium-89 is an approved medical radionuclide [71], a β-emitter with a half-life of 50.5 days, often administered as a chloride salt. Its uptake into osteoblastic bone metastases is *ca* fivefold greater compared to normal bone [72]. It is effective in palliative relief of pain from bone metastases, including breast and metastatic prostate cancer [73–75].

There is about 24 mg of *barium* (*Z*=56) in the body. It is not essential and often associated with toxicity. It is best known in medicine for its use in (relatively insoluble) BaSO_4_ meals administered as a radiopaque contrast agent for X-ray imaging of the gastrointestinal tract, providing radiographs of the oesophagus, stomach and duodenum. Barium sulfate has extremely low solubility in water (3.1 mg l^−1^) and is non-volatile making it unharmful to the body. A small amount of Ba^2+^ may be solubilized by gastric HCl. Barium in Brazil nuts is sometimes a concern. There is a report of a man consuming a single dose of 179 mg Ba from 92 g of Brazil nuts, with more than 91% of the dose absorbed [61].

All isotopes of *radium* (*Z*=88) are radioactive. There are ultra-trace amounts in our body with no beneficial role. Radium-223 dichloride is an α-emitting pharmaceutical (half-life 11.4 days) approved for treatment of symptomatic bone metastases and castration-resistant prostate cancer (http://www.xofigo-us.com/index.php (accessed 10 July 2014)). ^223^Ra^2+^ is a Ca^2+^-mimetic that selectively targets bone metastases with short-range high-energy *α* particles which cause double-strand breaks in DNA as well as greatly localized cytotoxicity, with negligible myelosuppression [76–80]. The activity of ^223^Ra *in vivo* has prompted evaluation of its efficiency and safety in clinical trials of patients suffering from bone-metastatic prostate cancer [77,79,81–84]. It is an effective candidate for palliation of bone pain.

## 4. Groups 3–12: the transitions metals

### (a) First transition series

*Scandium* (*Z*=21) is not an essential element, but its radioisotopes have potential in PET and SPECT imaging, as well as therapy [85]. Both ^44^Sc (*t*_1/2_=3.93 h, 94% β^+^, 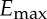 1474 keV) and ^47^Sc (*t*_1/2_=3.35 days, 100% β^−^, 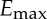 162 keV) display appropriate properties for diagnosis or therapy, and, in combination, can be used for theranostic applications. ^44^Sc is a candidate for PET imaging using radiometalated peptides or other small targeting biomolecules [86]. Preclinical evaluations of DOTA-functionalized biomolecules radiolabelled with ^44^Sc have demonstrated effectiveness for PET imaging [87]. ^44^Sc could be a useful radioisotope for clinical nuclear imaging and pre-therapeutic dosimetry of cancer patients prior to treatment with a therapeutic ^177^Lu-labelled DOTA derivative. ^47^Sc being a β-emitter has potential as a therapeutic radionuclide and combined with ^44^Sc would allow use of matching radiopharmaceuticals with the same pharmacokinetics [87].

**Figure 5.**
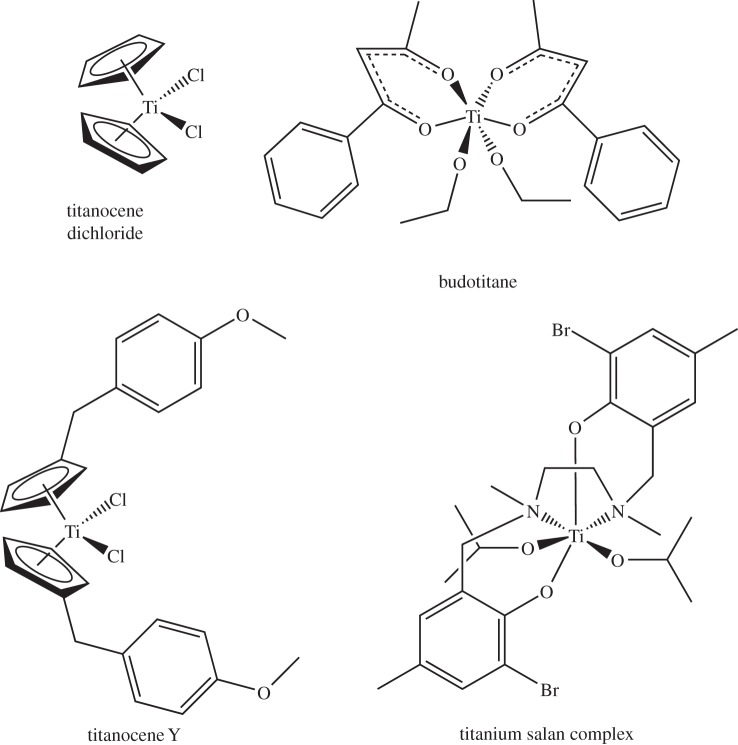
Titanium complexes with anti-tumour properties. Titanocene dichloride and budotitane both underwent clinical trials but were abandoned because of instability in aqueous media.

*Titanium* (*Z*=22) is not thought to be essential and is best known in medicine as a light, strong metal in implants, and in anti-cancer complexes, two of which have been on clinical trials. Mice given Ti^IV^ oxalate supplements show positive weight gains and a reduction in tumour development [88]. Beneficial effects on other animals have also been reported [89,90], and interestingly titanium compounds, patented as fodder additives, are claimed to improve weight gain in domestic animals [91]. Titanium(IV) complexes were the first class of metal compounds to enter clinical trials after platinum complexes for the treatment of cancer [92]. Budotitane and titanocene dichloride (figure 5) exhibit anti-tumour activity and low toxicity in several cancer cell lines [93]. Unfortunately, clinical trials of these compounds initiated in the 1980s were finally abandoned due their instability in water [92,94]. Since then, other Ti^IV^ complexes have been developed with the aim of overcoming this instability. Among these, titanocene derivatives and titanium salan complexes show promise [95,96]. The titanium complexes Ti-Salan and Ti-Y display contrasting behaviour regarding their reactivities with DNA or albumin, their cellular uptake and intracellular distribution [97]. Ti-Salan shows relatively low binding to biomolecules but increased serum-dependent cellular uptake whium salts are widely used for treatment of bipolar disorders (BDs). Li^+^ is a very small ion (6-coordinate radius 0.76 Å, figure 1) with a high hydration enthal

Interest in the essentiality of *vanadium* (*Z*=23) was aroused by the finding about 35 years ago that vanadate present as an impurity in commercial horse skeletal muscle ATP inhibits the enzyme Na^+^/K^+^-ATPase [98]. Vanadium is thought to be essential for man, but its roles in the body (total of *ca* 2.4 mg) are poorly understood. Vanadium complexes have been on clinical trials recently as antidiabetic agents.

Genes that code for V are known in some organisms. For example nitrogen-fixing bacteria such as *Azotobacter* [99] contain a Fe_7_VS_9_ *M*-cluster in vanadium nitrogenase with vanadium coordinated to three sulfides, a histidine-N, and two oxygen functions of homocitrate. Hydrated V^III^ is present in vanadocytes (blood cells) of sea squirts (Ascidiacea) at a concentration of 350 mM! Mushrooms belonging to the genus *Amanita* contain amavadin where V^IV^ is coordinated to two tetradentate *N*-oxyimino-2,2′-dipropionate ligands. The *oxido*vanadium(V) H_2_VO core is found in vanadate-dependent haloperoxidases from, *inter alia*, marine algae, coordinated to an active centre histidine-N. These vanadate-dependent haloperoxidases and vanadium nitrogenases remain the only identified vanadium-containing enzymes in nature [99].

**Figure 6.**
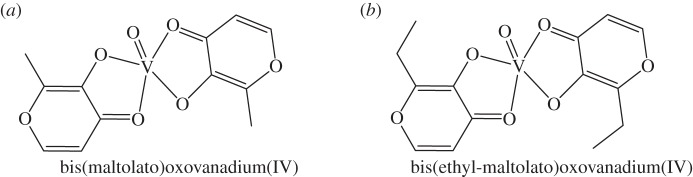
(*a*,*b*) Oxovanadium drugs of interest as insulin mimetics.

In vertebrates, particularly humans, V^IV^ and V^V^ are likely to predominate and the similarity between V^V^ as vanadate and phosphate is likely to be central to its biological effects. Vanadate-dependent haloperoxidases mimic enzymes involved in phosphate metabolism, where vanadate blocks the protein binding domain of phosphate. This competitive binding may account for the insulin-mimetic/insulin-enhancing potential of vanadium compounds. Vanadium complexes can be used to alleviate insufficient insulin response in diabetes mellitus [100]. They may not be able to completely make up for the lack of insulin (as in type 1 diabetes), but can lower dependence on exogenous insulin, or replace other oral hypoglycemic agents, in type 2 diabetes [101]. Both bis(maltolato)oxovanadium(IV) (BMOV) and the ethylmaltol analogue, bis(ethyl-maltolato)oxovanadium(IV) (BEOV) (figure 6), have undergone extensive pre-clinical testing for safety and efficacy [101]. BEOV has advanced to phase II clinical trials. These significant developments in vanadyl insulin mimetics have prompted further research into the biological applicability of vanadium complexes particularly for pharmacological control of cancer and diseases triggered by viruses, bacteria, amoebae and flagellate protozoan parasites [99,102].

In the late 1950s, *chromium* (*Z*=24) was proposed as an essential element. However there is no reliable current evidence that chromium is essential. No natural proteins in the body are known to contain Cr and there is no gene that can be said to code for it. Interest in the essentiality of chromium began with the isolation of a chromium complex from extracts of brewers' yeast which enhanced the action of insulin in controlling normal levels of blood sugar [103]. However this complex, so-called ‘glucose tolerance factor’, thought to contain Cr^III^ and nicotinate and glutathione as ligands, was never fully characterized. Moreover, the glucose tolerance factor itself was subsequently shown not to contain Cr [104].

There is still interest in the possible therapeutic effects of Cr, but its beneficial properties still remain tentative. Today, the US Food and Drug Administration (FDA) recommends a daily adult intake of about 30 μg Cr per day, despite its essentiality being in question. Vincent recently concluded that, in fact, Cr has been conclusively shown *not* to have beneficial effects on body mass or composition and should be removed from the list of essential trace elements [105].

Cr^III^tris(picolinate) is widely marketed as a mineral supplement for weightloss and body-building, although there is concern about possible damage to DNA [104]. Cr^VI^ (chromate) is known to be a genotoxic carcinogen. The toxicity seems likely to arise from redox reactions in cells which generate Cr^V^ 1,2-diolato species (carbohydrates, glycoproteins and sialic acid derivatives) which can cause oxidative damage to DNA [29]. A recent study of Cr^V^ complexes with a variety of monosaccharides and the model ligand *cis*-1,2-cyclopentanediol provided evidence of their nuclease activity [29]. The Cr^V^ complexes can cause oxidative DNA damage without the presence of added reductants or oxidants, supporting the participation of Cr^V^ 1,2-diolato complexes in the biological activities of both Cr^VI^ and Cr^III^.

There is no doubt that *manganese* (*Z*=25, total body Mn *ca* 16 mg) is essential, and that there are genetic codes for a range of Mn enzymes with functions in metabolism, reproduction, the immunological system, regulation of cellular energy and bone and connective tissue growth. Manganese as Mn^2+^ has chemistry similar to Mg^2+^, although unlike the latter it is redox-active, with the +3 state also being readily accessible, as in the enzyme mitochondrial manganese superoxide dismutase. The most abundant Mn-binding protein in the body is glutamine synthetase which plays a prominent role in brain chemistry (in astrocytes).

However, excessively high levels of Mn in the body can be toxic [104]. Industrial Mn toxicity is well recognized, as manganism, a neurodegenerative disorder. Manganism results from overexposure and production of reactive oxygen species and toxic metabolites as well as altered mitochondrial and ATP functions and exhaustion of cellular antioxidant defence mechanisms [106]. Mutations in the Mn exporter gene SLC30A10 are associated with motor impairment and Parkinson's disease-like symptoms.

An intriguing question is how does Mn cross the blood–brain barrier (BBB)? Despite several studies on the mechanism of transport of manganese across the BBB, the exact identity of the carrier is still unclear. Mechanisms using active transport [107] or facilitated diffusion [108,109] have been suggested, as well the high affinity metal transporters of calcium and iron. Manganese may also enter the brain via leak pathways in areas without an intact BBB [110]. Overall, it is likely that more than one transporter is responsible for transport of Mn across the BBB; several of them may work in a cooperative manner to maintain optimal Mn tissue concentrations.

There is about 4.8 g of *iron* (*Z*=26) in the body and the human genome codes for over 500 Fe-containing proteins [111]. In eukaryotes and prokaryotes, the level of non-haem iron proteins is relatively uniform and their relative number in the proteome decreases in passing from archaea (about 7%), to bacteria (about 4%), to eukaryotes (about 1%) [112]. Several genes are responsible for haem synthesis, haem transport and insertion of haem groups into haem proteins [111,113,114]. Particularly intriguing from an inorganic chemistry standpoint, is the presence of sulfide as a ligand in iron–sulfur proteins [115,116]. H_2_S itself is best known as a poison. Fe−S proteins are ubiquitous in cells and play many roles including electron transfer, catalysis and iron regulation (IRP proteins). Sulfide for Fe−S proteins is generated from cysteine by the enzyme cysteine desulfurase. Mutations in proteins involved in Fe−S cluster biogenesis are known to cause at least five distinctive human diseases. Iron–sulfur clusters are also associated with enzymes involved in DNA processing.

**Figure 7.**
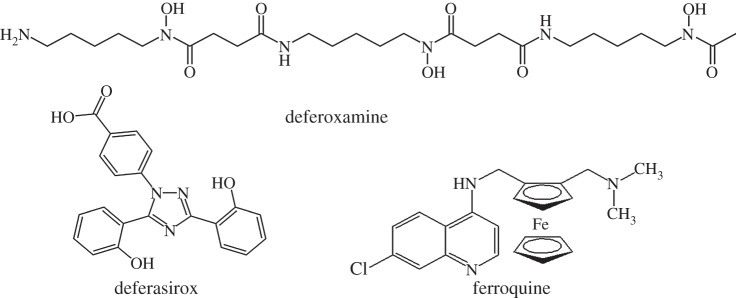
The injectable iron chelator deferoxamine, oral chelator deferasirox and antimalarial drug ferroquine.

The implications of overload or deficiency of iron have been discussed recently [114]. There is a need for new oral iron supplements—the most widely used supplement, ferrous sulfate, was introduced way back in 1832 as a treatment for anaemia. The bioavailability of Fe is affected by plant foods, including phytate (inositol hexaphosphate), polyphenols and tannins [117]. Elevated Fe levels seem to play a role in neurodegeneration [118]. In therapy, the Fe(II) containing compound ferroquine (figure 7), a ferrocene derivative, is currently in phase II clinical trials [119] for treatment of malaria. It is believed to exert its antiplasmodial properties via two mechanisms: interaction with free haem and generation of reactive oxygen species [120]. Ferrocifens, complexes containing ferrocene and tamoxifen fragments show promise as potential drugs for treatment of breast cancer [121]. Superparamagnetic iron oxides composed of nano-sized crystals are used as magnetic resonance imaging (MRI) contrast agents [122] and sodium nitroprusside, Na_2_[Fe^II^(CN)_5_NO], which slowly releases nitric oxide, has long been prescribed as a hypertensive agent [123]. Iron overload as in the inherited condition thalessaemia can be treated with iron chelating agents such as deferoxamine (desferrioxamine B, injectable) or more recently deferasirox (oral) (figure 7).

There is only *ca* 1.6 mg of *cobalt* (*Z*=27) in the body, but it plays a vital role in the coenzyme vitamin B12 (cobalamin), which has a recommended daily intake of 2–3 μg d^−1^. A deficiency of this vitamin results in pernicious anaemia through inactivation of one of two human enzymes for which B12 is the required coenzyme (methionine synthase and methylmalonyl-CoA mutase) [124]. Vitamin B12 can be synthesized only by microorganisms (bacteria and archaea). It contains cobalt tightly bound in a corrin ring and oxidation states +1, +2 and +3 are important in its biological activity. It was the first organometallic complex to be recognized in man and readily forms Co−C bonds (to deoxyadenosyl and methyl groups).

**Figure 8.**
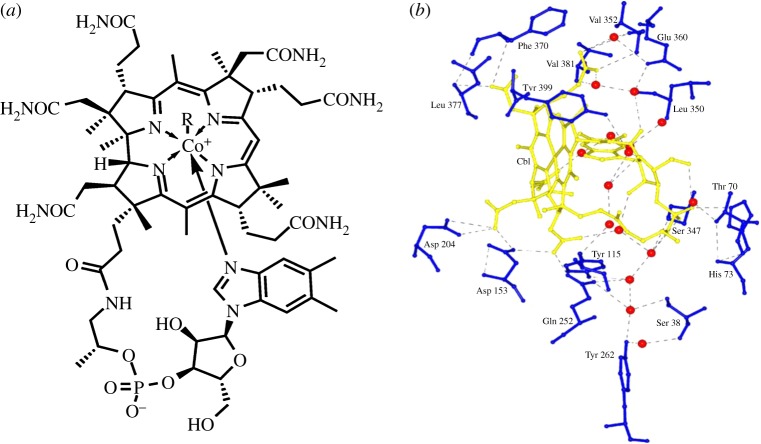
(*a*) Structure of vitamin B12, shown as a Co^III^ corrin complex, where *R*=5′-deoxyadenosyl Me, OH or CN (cyanocobalamin). (*b*) Environment around cobalamin (yellow) in the binding site of intrinsic factor, the glycoprotein produced in the stomach and responsible for absorption of vitamin B12 in the small intestine. Water molecules are shown as red spheres. Reprinted with permission from Mathews FS, Gordon MM, Chen Z, Rajashankar KR, Ealick SE, Alpers DH, Sukumar N. 2007 *Proc. Natl Acad. Sci. USA* **104**, 17 311–17 316.

Our genome codes for proteins in the digestive tract that selectively absorb B12 from the diet. Several gene products, including carrier proteins are involved in the absorption and distribution of vitamin B12 [125]. Altered cellular entry, transit or exit of the vitamin can result in deficiency and eventually give rise to haematological and neurological disorders [125]. Under physiological conditions, vitamin B12 is bound to the gastric intrinsic factor (figure 8) and is internalized in the ileum by a highly specific receptor complex composed of cubilin (Cubn) and amnionless (Amn) proteins [124,126]. Following exit of vitamin B12 from the ileum, general cellular uptake from the circulation requires the transcobalamin receptor CD320, whereas kidney reabsorption of cobalamin depends on megalin.

Perhaps surprisingly, there are few cobalt-based drugs. Doxovir, also known as CTC-96, is a Co^III^ bis(2-methylimidazole) acacen derivative which has completed clinical phase I trials for ophthalmic herpetic keratitis and adenoviral conjunctivitis and clinical phase II trials for herpes labialis [127]. We can expect Co^III^ to be reduced to the more labile Co^II^ inside cells.

There is *ca* 8 mg of *nickel* (*Z*=28) in the body, but it is not clear whether nickel is an essential element for man or not. Nickel allergy is certainly well known—one of the most common allergies in the world. Nickel is certainly an essential element for some bacteria.

**Figure 9.**
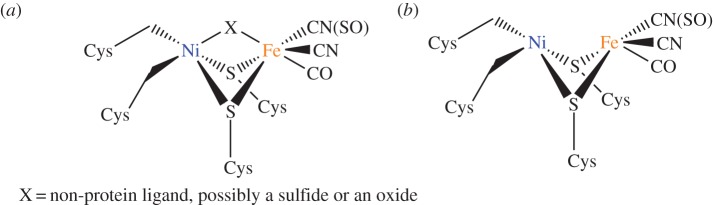
The nickel-containing active site in [NiFe] hydrogenases [128], a virulence factor for microorganisms such as *H. pylori*. These hydrogenases are classified into two categories, the oxidized form (*a*) which contains a non-protein bridging ligand and the reduced form (*b*) where the non-protein ligand is absent.

For vertebrates, no Ni-dependent enzymes are known [128]; however, in microorganisms several Ni-dependent enzymes have been well characterized. Owing to our symbiotic existence with microbes (of which there are 10× more than human cells in our body), it is possible that Ni is essential for the survival of some of the microbes on which we depend for survival [129]. However Ni can also make some microbes virulent. Nickel is a virulence determinant for the human gastric pathogen *Helicobacter pylori* which possesses two nickel enzymes that are crucial for *in vivo* colonization, [NiFe] hydrogenase (figure 9) and urease, with urease containing 24 nickel ions per active complex [130]. These two nickel trafficking partners in virulence are potential novel therapeutic targets for treatment of *H. pylori* infections which can prevent ulcers from healing [130].

As for beryllium (*vide supra*), metal hypersensitivity is also an immune disorder associated with Ni allergic hyper-reactivity [44]. Approximately 10% of the population suffers from Ni-contact dermatitis. CD4^+^ and CD8^+^ T cells have been identified as the major effector cells in nickel hypersensitivity [131]. The chemical basis of Ni sensitivity is also becoming better understood. Nickel(II) ions act as haptens that bind to the surface of the MHC and peptide complex, modifying the binding surface of MHC and triggering the immune response of T cells.

*Copper* (*Z*=29) is the third most abundant essential transition metal in the body, a total of *ca* 80 mg [132]. In eukaryotes and prokaryotes, the size of the copper proteome approaches 1% of the total proteome [133]. Copper is involved in important biological processes including respiration, angiogenesis and neuromodulation. The structural basis of the activity of copper enzymes and chaperones is now becoming well elucidated [134]. Copper proteins are classified as either type 1, 2 or 3 [135]. Type 1 (blue copper sites) function in single electron transfers [136]. In type 2 copper sites, copper acts as a catalytic centre and binds directly to substrates [135]. Type 3 copper sites are binuclear and involved in the activation and transport of oxygen [135]. Abnormalities in Cu homeostasis are thought to play a role in Alzheimer's, Parkinson's and motor neuron disease [137], but Scheiber *et al*. [134] point out that the full roles of Cu have yet to be clarified.

There is about 2.6 g of *zinc* (*Z*=30) in the body, and Zn^2+^ is involved in nearly all aspects of molecular and cell biology. Zinc proteins account for about 10% of the human proteome, *ca* 3000 zinc proteins with physiological functions [138]. Zinc plays a role in the structure of proteins as well as in enzymatic catalysis. Its coordination chemistry within proteins has been widely studied [139]. In some cells relatively high concentrations of zinc can be reached in vesicles (millimolar, especially in synaptic vesicles in the brain) where it is stored and undergoes controlled release [140]. With the recognized importance of Zn, Maret has stressed the need for more knowledge of interactions with biomolecules other than proteins if zinc biochemistry is to have a major impact on the diagnosis, prevention and treatment of human disease [140].

**Figure 10.**
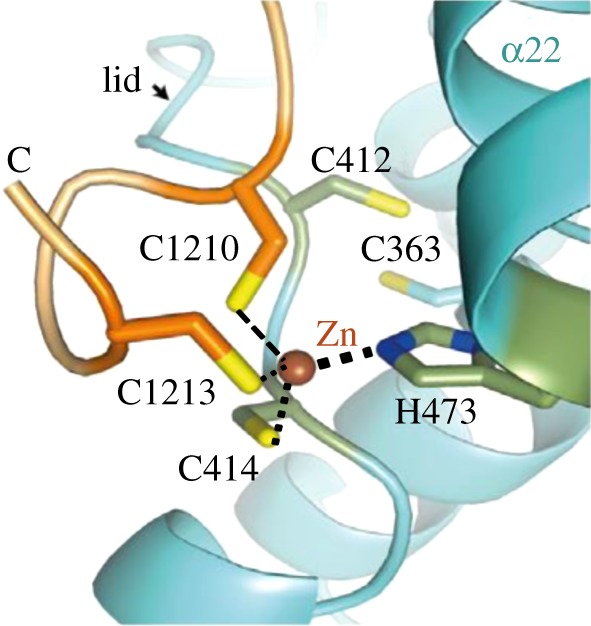
Zinc plays a role in controlling the mammalian circadian clock. The interfacial tetrahedral Zn binding site between mCRY1 (His473 and Cys414) and mPER2 (Cys1210 and Cys1213) which is involved in the regulation of clock genes. Adapted with permission from [142].

The functions of cells of all organisms are influenced by daily and seasonal changes due to the rotation of planets and their orbits around the sun. The predominantly light–dark cycle of the Earth's rotation gives rise to an endogenous circadian timing system that synchronizes biological functions [141]. Period (PER) proteins are essential to the mammalian circadian clock and recently the crystal structure of a complex comprising the photolyase homology region of mouse CRY1 (mCRY1) and a C-terminal mouse PER2 (mPER2) fragment revealed that zinc is involved in the stabilization of mCRY1–mPER2 interactions *in vivo* [142]. Figure 10 shows the zinc binding site [142].

### (b) Second and third transition series

**Figure 11.**
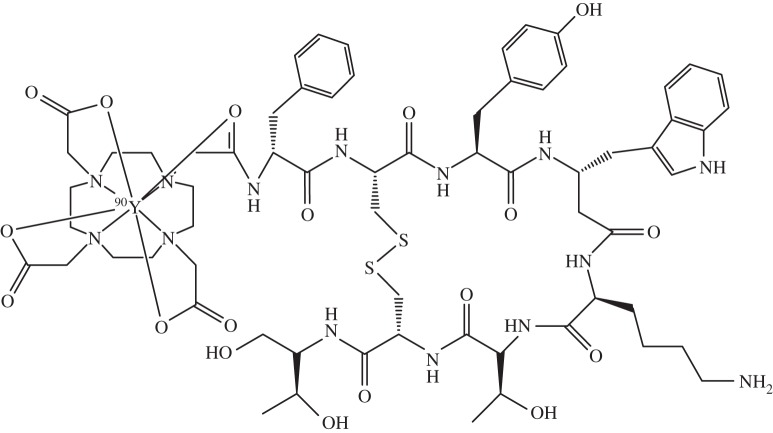
(^90^Y-DOTA-Phe-Tyr)octreotide [144]. ^90^Y^III^ is delivered to cells as a strongly chelated complex conjugated to a peptide vector (as shown here) or antibody where it can be imaged.

*Yttrium* (*Z*=39) is not an essential element, but is used clinically for cancer treatment as the radionuclide ^90^Y, half-life 2.7 days, a pure β-emitter [143]. It can be delivered to cells as a strongly chelated complex, e.g. (^90^Y-DOTA-Phe-Tyr)octreotide (SMT487, DOTATOC), figure 11, targeted to somatostatin receptors (SSTRs). ^86^Y, half-life 14.7 h, which decays by electron capture, can play a complementary role to ^90^Y for PET imaging of the *in vivo* biodistribution and dosimetry of therapeutic ^90^Y pharmaceuticals [144].

An average (80 kg) man contains *ca* 4 mg of *zirconium* (*Z*=40), but Zr has no known biological role. We ingest *ca* 4.2 mg d^−1^ depending on dietary habits [145]. Use of zirconium for biomedical applications is steadily growing [146], e.g. in dental implants [147,148], total knee and hip replacements [149] and middle-ear ossicular chain reconstruction surgery [150]. Like Al^III^, Zr^IV^ readily forms polymeric oxygen-bridged complexes (e.g. Zr–O(H)–Zr bridges) and is widely used in antiperspirants to coat the skin and prevent escape of (bacterial) body odours [151]. An example is the glycine, chlorido, hydroxido substance aluminium zirconium tetrachlorohydrex gly (as named by the International Nomenclature of Cosmetic Ingredients).

*Niobium* (*Z*=41) is not essential and has yet to be explored widely in therapy. Polyoxometalates containing Nb^V^ are of interest as potential antiviral agents, e.g. heteropolyniobate [SiW_9_Nb_3_O_40_]^7−^ [152].

**Figure 12.**
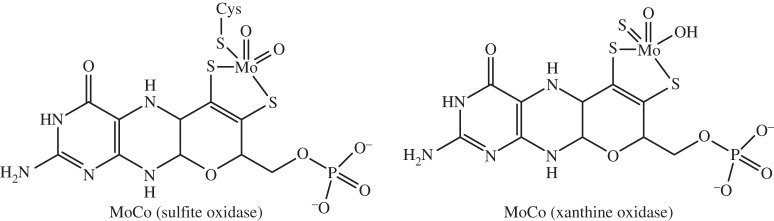
Structures of the cofactor MoCo in xanthine oxidase and sulfite oxidase [153].

*Molybdenum* (*Z*=42) is the only essential trace element in the second and third transition series. Of the *ca* 8 mg in the body, the highest concentrations are in the kidney, liver, small intestine and adrenals [153,154]. The only known chemical form of Mo taken up by cells is the oxyanion molybdate, [Mo^VI^O_4_]^2−^. The human genome codes for four Mo enzymes, in the xanthine oxidoreductase and sulfite oxidase families, in which Mo is bound at the active site by a special molybdenum cofactor (molybdopterin, MoCo, figure 12) [155].

MoCo cofactor deficiency due to gene mutations is known, and can lead to rapid neurodegeneration [156]. In these Mo enzymes, the transfer of oxygen to or from substrates is catalysed by Mo using water as an oxygen atom donor or acceptor. Molybdenum interconverts between two oxidation states, Mo^IV^ and Mo^VI^. MoCo is synthesized using a highly conserved multi-step biosynthetic pathway. If this biosynthesis is deficient, then a pleitropic loss of all four human Mo-enzyme activities can occur, and in most cases is accompanied by early childhood death [153].

Molybdate [MoO_4_]^2−^ is reported to prevent oxidation of lipids and protect antioxidant systems in experimental diabetic rats, and thus may be useful for treatment of diabetic mellitus [157]. Tetrathiomolybdate [MoS_4_]^2−^ is a copper chelator of interest in medicine not only for its ability to lower copper levels [158], but also for treatment of breast cancer and oesophageal carcinoma for which it is in phase II clinical trials.

**Figure 13.**
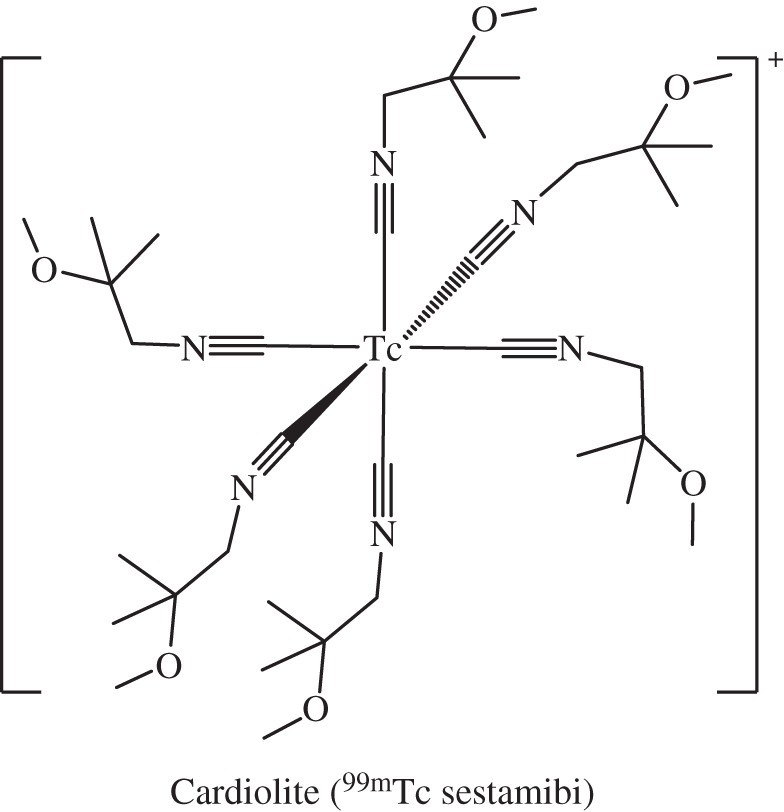
The ^99m^Tc^I^ complex used for SPECT (*γ*-ray) imaging of heart muscle. The complex was discovered in the laboratory of Alan Davison at MIT.

*Technetium* (*Z*=43) is man-made, dating back to 1937. The metastable radioisotope ^99m^Tc, a γ-emitter with half-life of 6 h, is used in tens of millions of single photon emission computed tomography (SPECT) diagnostic procedures every year. It is readily generated at the bedside from the longer lived isotope ^99^Mo. More than 50 ^99m^Tc radiopharmaceuticals are currently in use for imaging and functional studies of various areas of the body, including bone, the brain, thyroid, lungs, myocardium and liver [159]. The ligands play a crucial role in the targeting properties of the complexes, e.g. phosphonate and phosphate complexes for bone. By way of current examples, Cardiolite (^99m^Tc-sestamibi, figure 13) and Neurite (^99*m*^Tc-disicate) have been approved for folate-receptor positive tumours [160]. ^99m^Tc-MIP-1404 is in clinical phase II trials for prostate cancer imaging [161,162].

**Figure 14.**
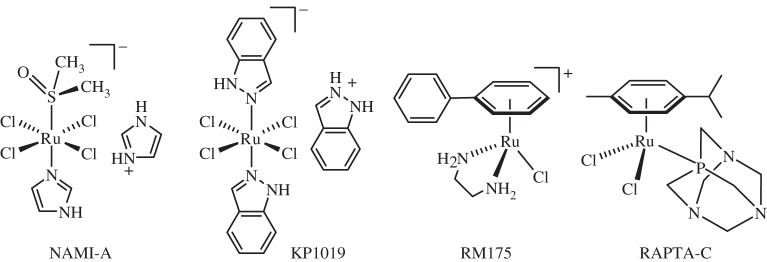
Some Ru^III^ and Ru^II^ anti-cancer complexes. Both NAMI-A and KP1019 (as the sodium salt NKP-1339 and IT-139) are in clinical trials.

Two *ruthenium* (*Z*=44) complexes are in clinical trials as anti-cancer drugs. They are both octahedral Ru(III) complexes. The indazole complex KP1019, *trans*-[RuCl_4_(In)_2_]InH (under commercial clinical development as the sodium salt NKP-1339 and IT-139, figure 14) is more cytotoxic to the primary tumour cells than the imidazole complex *trans*-[RuCl_4_(Im)(DMSO)]ImH, NAMI (new anti-tumour metastasis inhibitor)A, which is active against metastases [161,163]. Both have completed phase I clinical trials [164–169]. Ruthenium(III) from these complexes may be delivered to tumour cells by the Fe^III^ transport protein serum transferrin, receptors for which are over-expressed on cancer cells. Once in cells the Ru^III^ may be activated by reduction to Ru^II^, although Ru^IV^ is also accessible under biological conditions. KP1019 induces apoptosis via the intrinsic mitochondrial pathway. Organometallic Ru^II^ arene complexes also exhibit promising anti-cancer activity [170] and Ru^III^ EDTA complexes have been investigated as NO scavengers for treatment of septic shock [171].

Complexes of the rare precious metal *rhodium* (*Z*=45) are currently the focus of several studies as anti-tumour, antiparasitic and antiviral agents [172–175]. The radionuclide ^105^Rh (β^−^ emitter, half-life 35.4 h) is useful medically. For example, ^105^Rh-EDTMP is a promising therapeutic agent for treatment of pain due to bone metastases, displaying rapid blood clearance and selective uptake into bone [176].

As yet, *palladium* (*Z*=46) has found only limited uses in medicine. A Pd-bacteriopheophorbide (TOOKAD) is a photoactivatable compound that is currently being evaluated in phase III clinical trials for localized prostate cancer and in phase I/II trials for treatment of small renal tumour targets [177,178]. It can be activated by long wavelength light (approx. 763 nm) allowing deep tissue penetration and displaying rapid clearance from the bloodstream [177].

The radionuclide ^103^Pd (half-life 17 days, decays by electron capture) was introduced for brachytherapy in 1989 [179]. A study spanning 11 years found that utilization of ^103^Pd for plaque radiotherapy of choroidal melanoma resulted in improved visual function when compared to the use of ^125^I [180]. Currently, ^103^Pd is in phase III clinical trials for treatment of early stage prostate cancer [181,182].

*Silver* (*Z*=47) is best known in daily life for its potent antimicrobial properties. As far back as the eighteenth century silver was used for wound management [183]. In the form of silver nitrate, it was employed to treat ulcers [184] and silver ions were identified as antimicrobial in the nineteenth century. Colloidal silver was approved for wound management by the FDA in the 1920s [185]. In 1968, silver nitrate was combined with a sulfonamide antibiotic to produce silver sulfadiazine, a topical antibacterial agent prescribed for burn management that is still in use [186,187]. Silver-containing wound dressings are often used in lieu of prescription antibiotics due to the emergence and rise in (organic) antibiotic-resistant bacteria [188]. Acticoat absorbant is a silver-releasing dressing in phase IV clinical trial for the prevention of lower extremity revascularization wound complications [189]. Silver nanoparticles are also being evaluated as antiviral agents [190] and for drug delivery [191].

There is *cadmium* (*Z*=48) in the body (*ca* 56 mg) and although it is possible that it is essential at very low doses (see Introduction), it is usually considered to be toxic. Cadmium(II) has a high affinity for sulfur ligands, e.g. in the protein metallothionein, where it can displace natural Zn^2+^ from Zn(Cys)_4_ sites [192], which may have implications for the cellular toxicity of Cd [193]. Intriguingly, the cadmium-dependent carbonic anhydrase in the marine diatom *T. weissflogii* [194] uses Cd as its natural metal cofactor when only low concentrations of Zn are available.

*Hafnium* (*Z*=49) has no known biological role. There is interest in 50 nm diameter spheres of hafnium oxide functionalized with a negative surface (NBTXR3) [195,196]. NBTXR3 nanoparticles are taken up efficiently by tumour cells and can enhance the effects of radiation therapy. They are in phase I clinical development for advanced soft tissue sarcomas and head and neck cancer [197].

*Tantalum* (*Z*=73) is best known for its inertness and hardness as a metal and is commonly used in implants and bone repair [198,199]. The porosity of Ta provides a scaffold for bone ingrowth and mechanical attachment [200–202].

*Tungsten* (*Z*=74) is not essential for man. It is most commonly encountered in biological systems as W^VI^ in tungstate [WO_4_]^2−^ [203]. Sodium tungstate has antidiabetic properties. [WO_4_]^2−^ can normalize glycemia when administered orally in several types 1 and 2 diabetic animal models [204–207]. In primary cultured hepatocytes sodium tungstate behaves in a similar manner to insulin, increasing glycogen synthesis and accumulation [208], and induces a transient strong activation of extracellular signal-regulated kinases 1 and 2 (ERK1/2), in the same way as insulin. Some oxidoreductase enzymes in bacteria employ W in a similar manner to Mo [209,210]. Polyoxotungstates are also being studied for a range of other medicinal applications including antiviral [211], antibacterial [212], anti-cancer [213] and for the treatment of Alzheimer's disease [192].

Radioisotopes of *rhenium* (*Z*=75), ^188^Re (half-life 16.9 d, β^−^ emitter) and ^186^Re (half-life 3.8 days, decays by electron capture and β^−^ emission), are used to treat cancer. Colloidal sulfur particles labelled with ^186^Re are used in radiation synovectomy in the treatment of rheumatoid arthritis [214] and ^188^Re-1,1-hydroxyethylidenediphosphonate (^188^Re-HEDP) for bone pain palliation in patients suffering prostate cancer [215]. ^188^Re P2045 is currently in phase I/II clinical trials for treatment of small cell lung cancer and other advanced neuroendocrine carcinomas [216]. P2045 is an 11-amino acid somatostatin analogue peptide which has a high affinity for the SSTR [217]. The SSTR is expressed in both small and non-small cell lung cancers [218,219] as well as on peritumoural blood vessels in numerous malignancies [220,221].

The only current medical use of the precious metal *osmium* (*Z*=76) is injections of aqueous osmium tetroxide (OsO_4_) to destroy diseased tissue in chronically inflamed arthritic joints [222,223]. OsO_4_ acts as a superoxide dismutase mimic, catalysing the dismutation of the superoxide which is a primary inflammatory species [224]. Organo-osmium arene complexes show promise as anti-cancer drugs [225].

**Figure 15.**
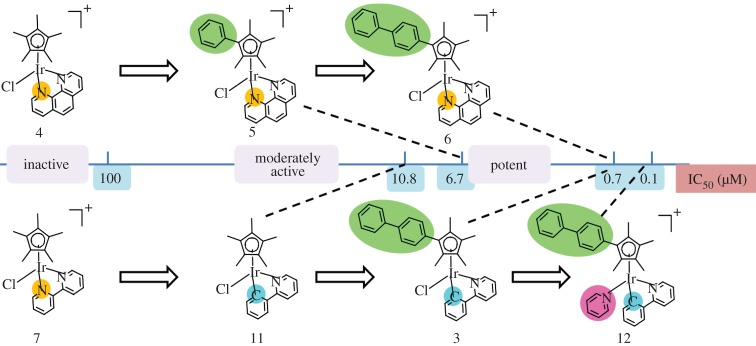
Structure–activity relationships for half-sandwich Ir^III^ cyclopentadienyl anti-cancer complexes towards A2780 human ovarian cancer cells [231]. Reprinted with permission from Liu Z, Sadler PJ. 2014 *Acc. Chem. Res.* **47**, 1174–1185. Copyright • 2014 American Chemical Society.

*Iridium* (*Z*=77) is used clinically for cancer brachytherapy as the radionuclide ^192^Ir (half-life 73.8 days, β^−^ emitter), and is currently in phase III evaluation for stage B2 and C prostatic carcinoma [226] and in phase I/II trials of a GAMMA-Iridium-192 catheter for coronary artery disease [227]. Iridium(III) complexes can act as inhibitors of protein kinases and protein–protein interactions [228], and photoactive polypyridyl iridium complexes are potential candidates for photodynamic therapy [229–231]. Organoiridium(III) cyclopentadienyl complexes show promise as anti-cancer agents (figure 15). Some appear to catalyse the conversion of NADH to NAD^+^ in cells by transfer of hydride to Ir^III^ [231].

**Figure 16.**
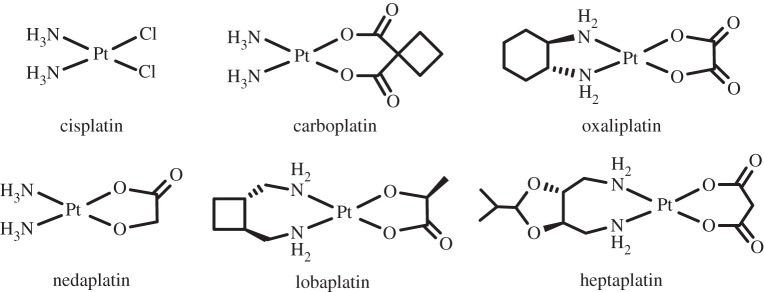
Platinum anti-cancer complexes which have been approved for clinical use [232].

Currently the most widely used drugs for cancer chemotherapy are *platinum* (*Z*=78) complexes, now components of approaching 50% of all treatments. The initial drug cisplatin *cis*-[PtCl_2_(NH_3_)_2_] (figure 16) originated from the laboratory of Barnett Rosenberg at Michigan State University in the 1960s, and has now been joined by its analogues carboplatin *cis*-[Pt (1,1-dicarboxycyclobutane)(NH_3_)_2_] and oxaliplatin [Pt(1*R*,2*R*-1,2-diaminocyclohexane)(oxalate)] which have been approved worldwide. Nedaplatin, lobaplatin and heptaplatin are approved for use in Japan, China and South Korea [233]. Lipoplatin, formed from cisplatin and liposomes of dipaomitoyl phosphatidyl, soy phosphatidyl choline (SPC-3), cholesterol and methoxypolyethylene glycol-distearoyl phosphatidylethanolamine (mPEG2000-DSPE) [234] has completed phase III clinical trials [234,235]. The highly positively charged Pt_2_ and Pt_3_ complexes of Farrell form an interesting new class of potent agents [236].

There is interest in nanoparticle formulations of platinum drugs that can include built-in targeting vectors [237,238]. The primary cellular target of platinum drugs is DNA [239], but there is increasing interest in the role of proteins, especially the copper transporter CTR1 [240]. Platinum(IV) complexes with their low-spin d^6^ configuration are of interest as pro-drugs which can be reduced *in vivo* either chemically (e.g. by thiols or ascorbate) or by irradiation with light to active Pt^II^ species [238,241,242]. Polymer nanoparticles that contain a covalently linked Pt^IV^ polymer conjugate are stable during circulation, enter target cells by endocytosis, and rapid intracellular drug release is triggered by the acidic environment [243].

**Figure 17.**
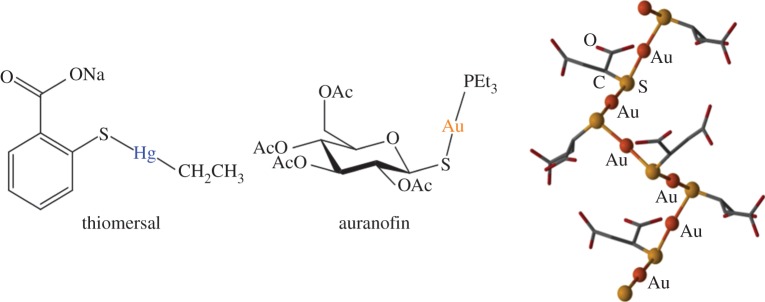
The antibacterial Hg^II^ agent thiomersal, oral antiarthritic Au^I^ drug auranofin and polymeric structure of the injectable antiarthritic Au^I^ drug aurothiomalate (Myocrisin).

The use of *gold* (*Z*=79) in medicine (chrysotherapy) dates back to ancient times, when Chinese and Indian practitioners used powered gold to treat many illnesses including arthritis [244]. The modern application of gold compounds in medicine was prompted by Robert Koch's discovery of the tuberculostatic properties of gold cyanide solutions [244,245], and by the (mistaken) belief by the French physician Forrestier that tuberculosis and rheumatoid arthritis are linked. From the 1930s, gold(I) complexes including Na_3_[Au(S_2_O_3_)_2_] (Sanocrysin), aurothiomalate (Myocrisin), and aurothioglucose (Solganol) have been administered by intramuscular injection, and in 1986 these were joined by the oral drug auranofin (figure 17), (triethylphosphine)gold(I)tetra-acetylthioglucose) (Ridaura) [244,245]. Aurothiomalate and aurothioglucose are polymeric with thiolate S atoms bridging Au(I) ions giving linear S–Au–S coordination. The target sites for gold drugs are likely to be proteins, especially cysteine thiolate sulfur and selenium in selenocysteine, as in mitochondrial thioredoxin reductase (TrxR) [244].

There is interest in using auranofin for other types of therapy such as anti-HIV, chronic lymphatic leukaemia and squamous cell lung cancer [245,246]. The potential of gold nanoparticles (AuNPs) is now being explored with many examples of AuNPs conjugated to peptides or antibodies being studied as gene-regulating agents, drug carriers, photothermal therapy agents and imaging agents [247–250]. Recently, the activity of auranofin against amoebic dysentery has been discovered [251].

These days, the use of *mercury* (*Z*=80) in therapeutics is declining. In earlier times, Hg was used to treat syphilis, as an antibiotic and was the active ingredient in diuretics, antiseptics, analgesics and laxatives [252]. Dental amalgam fillings are about 40% Hg by weight. Thiomersal, an organomercury compound, has antiseptic and antifungal properties. It was mainly used as an antimicrobial preservative in vaccines in the mid-twentieth century to prevent adverse effects such as *Staphylococcus* infection [253]. It did not reduce the potency of the vaccines [254]. There is a fear that thiomersal could trigger or cause autism in children, but this has yet to be proven [255]. Some vaccines not recommended for young children do still contain thiomersal such as those prescribed for diphtheria and tetanus (http://www.who.int/vaccine_safety/committee/topics/thiomersal/questions/en/ (accessed 11 August 2014)), and the World Health Organisation has deemed that there is no solid evidence of toxicity from thiomersal (http://www.who.int/biologicals/areas/vaccines/thiomersal/en/ (accessed 11 August 2014)).

### (c) The lanthanide and actinide elements (group 3+)

*Lanthanum* (*Z*=57) in the form of La_2_CO_3_ (Fosrenol) was approved in 2004 for treatment of hyperphosphatemia [256], an electrolyte imbalance that results in elevated levels of phosphate in the blood. Hyperphosphatemia is more severe in patients suffering from renal failure as a result of increased FGF-23 levels, secondary hyperparathyroidism and enhanced progressive vascular calcification [257]. Fosrenol acts as a phosphate binder, reducing absorption of phosphate through the formation of insoluble lanthanum phosphate complexes which can then pass through the grastrointestinal tract unabsorbed [258]. *In vitro* experiments show that La^3+^ binds to phosphate in the pH range of 3–7. Approximately 97% of available phosphate is bound between pH 3 and 5 and 67% at pH 7 in simulated gastric fluid with La^3+^ in a twofold molar excess over phosphate (http://www.rxlist.com/fosrenol-drug/clinical-pharmacology.html (accessed 11 August 2014)).

Interestingly, lanthanum, cerium and praseodymium compounds have been used as feed additives for animal production in China for more than 50 years [259]. It is well documented that small amounts of these additives can lead to greater weight gain in pigs, cattle, sheep and chickens [259,260].

*Cerium* (*Z*=58) has bacteriostatic properties; it is effective in the form cerium nitrate against a wide range of bacteria in a pH-dependent manner. In *Escherichia coli*, Ce is readily taken up into the cell cytoplasm, in contrast to mammalian cells, and there is clear inhibition of cellular respiration, glucose metabolism and oxygen uptake [261]. A Ce^3+^ nitrate–silver sulfadiazine formulation (also known as Flammacerium) has been used for treatment of burn wounds [262]. Cerium nitrate is believed to exert a protecting effect against postburn immunosuppression caused by a high molecular weight (3 MDa) lipid protein complex [262–265].

The *samarium* (*Z*=62) radionuclide ^153^Sm (β^−^ decay, half-life 1.9 days) is one of the more extensively used radiopharmaceuticals and as ^153^Sm-ethylenediaminetetramethylphosphonic acid (^153^Sm-EDTMP) stabilizes pain in patients suffering from osteoblastic metastatic bone lesions [266]. ^153^Sm-EDTMP is well tolerated by the body at doses of 37 MBq kg^−1^ body weight [266], shows an affinity for skeletal tissue and localizes in the skeleton and areas of increased bone turnover [267–269]. Subsequent to intravenous dosing, *ca* 50–60% of this complex is concentrated in bone within 2–3 h of administration [270], the highest amount reported for bone-seeking radiopharmaceuticals [271]. It has a small range of emission in bone (1.7 mm), thus limiting exposure of bone marrow and adjacent tissues to radiation [269,272]. ^153^Sm-EDTMP is currently in phase II clinical trials for treatment of high-risk osteosarcoma [273] and breast cancer metastatic to bone only [274] as well as phase I/II trials in combination with zoledronic acid or pamidronate for patients suffering from relapsed or refractory multiple myeloma and bone pain [275].

There are only a few examples of the use of *europium* (*Z*=63) in medicine. It has potential as a PARACEST MRI contrast agent. Paramagnetic metal complexes are often employed as exogenous contrast agents to reduce the relaxation time of water protons, thus enhancing tissue contrast in MRI [276]. A europium(III) DOTA-tetraamide complex can act as an MRI sensor of singlet oxygen (^1^O_2_) [276]. This complex forms an endoperoxide derivative upon rapid reaction with ^1^O_2_ resulting in a *ca* 3 ppm shift of the Eu^3+^-bound water peak through chemical exchange saturation transfer. This could prove useful for detection of singlet oxygen in cells during photodynamic therapy. Luminescent Eu^3+^-doped nanoporous silica nanospheres functionalized with folate *N*-hydroxysuccinimidyl ester molecules have been designed to enhance the imaging of cancer cells [277].

**Figure 18.**
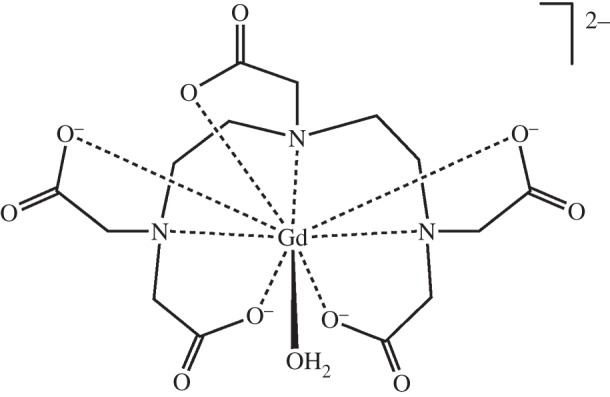
The intravenous MRI contrast agent [Gd(DTPA)(H_2_O)]^2−^. The paramagnetic 4f^7^ Gd^3+^ ion is large (ionic radius 1.2 Å) and can accommodate eight coordinating atoms from the chelating DTPA ligand and a water molecule. Exchange of coordinated water with bulk water is important for producing contrast in the magnetic resonance image.

Millions of doses of *gadolinium* (*Z*=64) are now administered every year as contrast agents for MRI. Gadolinum(III) with its seven unpaired electrons and slow electronic relaxation time is effective for relaxing H_2_O protons which can give rise to contrast in MR images. Thanks to the high thermodynamic and kinetic stability of chelated complexes such as [Gd(DTPA)(H_2_O)]^2−^ (figure 18, approved for clinical use in 1988 as gadopentetate dimeglumine, Magnevist) [278] and [Gd(DOTA)(H_2_O)]^−^ (Dotarem), they can be safely injected in gram quantities. The large Gd^III^ ion (radius *ca* 1.2 Å) can accommodate eight coordinating atoms from the chelating ligand plus a water ligand which exchanges rapidly with bulk water. There is concern about possible side-effects arising from any release of Gd(III) (e.g. displacement by Ca(II)) in the body, especially for less stable contrast agents. Currently, phase IV clinical trials are underway to evaluate Gd retention in the bones of patients with impaired renal function [279].

The radioisotope of *holmium* (*Z*=67) ^166^Ho is used as a substitute for ^188^Re (β^−^ decay, half-life 19.4 h) due to its suitable characteristics for internal radiation therapy. ^166^Ho has a half-life of 26.8 h, and emits high β-energy and low γ-energy which can be easily detected by gamma cameras [280]. Chitosan functionalized with ^166^Ho is being used as a cancer radiopharmaceutical in Korea [281]. Poly(l-lactic acid) microspheres loaded with ^166^Ho-acetylacetonate are being used for intra-arterial radioembolization in patients with unresectable liver malignancies [282,283]. These microspheres have already completed phase I trials for treatment of liver metastases [284] and will begin phase II trials in unresectable liver metastases soon [285].

The *ytterbium* (*Z*=70) radioisotope ^175^Yb also displays favourable decay characteristics for therapeutic applications, with a half-life of 4.2 days and a maximum β-energy of 480 keV [286]. ^175^Yb-labelled polyaminophosphonates have been evaluated for palliative therapy of bone metastases [287]. These complexes have high bone uptake with a minimal uptake in soft tissue and rapid blood clearance. Ytterbium-169 is a γ-emitter with a much longer half-life of 32 days, and is being explored as a potential therapeutic for intravascular brachytherapy [288].

The ^177^Lu radionuclide of *lutetium* (*Z*=71), half-life 6.7 days, with its β and *γ* emissions can be used for both therapy and SPECT imaging [289,290]. Two radiolabelled anti-epidermal growth factor receptor (EGFR) antibodies have been assessed for their EGFR-targeting and radioimmunotherapy efficacy [289]. Both display good tumour uptake and delay tumour growth significantly. Pretargeted radioimmunotherapy combining ^177^Lu-IMP-288 peptide and antibodies can deliver a higher radiation dose to tumours than a directly labelled antibody and is currently in clinical phase I/II trials for treatment of small cell lung cancer [291].

The radioisotope ^225^Ac of *actinium* (*Z*=89) which decays by β^−^, *α* and β^+^ (electron capture) emissions with a half-life of 10 days, is a useful radiopharmaceutical. Its cascade decay products include ^213^Bi, also an *α* (and β^−^) emitter useful for targeted radiotherapy [292]. Alpha-emitters are appropriate for management of minimal disease, including haematologic cancers, infections and compartmental cancers like ovarian cancer [293]. Lintusumab-Ac225 is about to enter phase I/II clinical trials in combination with Cytarabine for older patients with untreated acute myeloid leukaemia (AML) [294] and actinium-225-labelled humanized anti-CD33 monoclonal antibody HuM195 is completing phase I trials for patients with advanced myeloid malignancies [295].

## 5. Group 13: the boron family

As yet there is no convincing evidence that *boron* (*Z*=5) is an essential element for man. In contrast boron is thought to be essential for the growth and development of vascular plants, diatoms, marine algae and cyanobacteria [296–298]. It may play a role in the development of healthy bones and joints, and as a dietary supplement might be effective in preventing or treating arthritis [299,300]. Boron can stabilize and extend the half-life of vitamin D and estrogen, both of which are essential to bone health [300].

**Figure 19.**
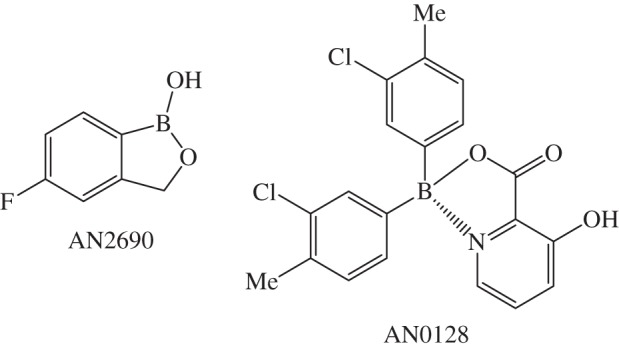
Two boron-containing drugs on clinical trial [305].

Boromycin is a polyether-macroclide antibiotic isolated from the Gram-positive *Streptomyces antibioticus* bacteria. It kills Gram-positive bacteria by adversely affecting the cytoplasmic membrane resulting in loss of potassium ions from the cell [301,302]. *In vitro* studies suggest that boromycin can inhibit replication of the clinically isolated HIV-1 strain and it may exert its anti-HIV activity by blocking later stage infection and the maturity step needed for replication of HIV [303]. Boron-containing compounds are in development as therapeutics that target not only microbial or neoplastic systems but cell-signalling processes that are intrinsic to many disorders [304]. Anacor Pharmaceuticals currently has several boron-containing compounds in development. Borinic picolinate (AN0128, figure 19) is a boronic acid ester with both anti-microbial and anti-inflammatory activity for acne and mild to moderate atopic dermatitis (http://www.anacor.com/pdf/AAD_P108.pdf (accessed 10 November 2014)) [306]. AN2690 (Kerydin) is an oxaborale topical antifungal approved by the FDA for treatment of onychomycosis of the toenails as a result of *Trichophyton rubrum* or *Trichophyton mentagrophytes* (http://www.anacor.com/pdf/Kerydin%20labeling.pdf (accessed 10 November 2014)).

*Aluminium* (*Z*=13) is the most abundant metal by weight in the Earth's crust and the third most abundant element after oxygen and silicon. It has no known role in human biology but there is evidence that Al may be linked to acute and chronic diseases in humans [307–309]. However problems seem to arise only in cases of kidney failure or if the kidney is underdeveloped (babies). Then Al absorption into the blood can lead to Al deposition in the brain, to dementia and even death [309]. Aluminium is present in many commercial products, including cosmetics, and Al exposure is rising [308].

Aluminium may impair mitochondrial bioenergetics via generation of reactive oxygen species which in turn leads to oxidative stress. Oxidative stress has been implicated as a factor in certain neurodegenerative conditions such as Alzheimer's disease and Parkinson's disease [307]. Aluminium salts are used as adjuvants in vaccines. Adjuvants are believed to promote immune responses either through recruitment of antigen professional antigen-presenting cells (APCs) to the vaccination site for optimal delivery of antigens to APCs or by activation of APCs to make cytokines and prompting T-cell response [310]. Aluminium salts act by activating the nucleotide binding domain-like receptor protein-3 (NLRP3) via one of two models of phagocytosis [311]. Aluminium salts are still in use as adjuvants (table 1). Alum (hydrated potassium aluminium sulfate), while one of the oldest and most common adjuvants used, is now being reconsidered in vaccine formulations as there are questions about its toxicity [312,313].

**Table 1.** Aluminium salts still in use as adjuvants [310].

aluminium salt	vaccine
aluminium phosphate	DTaP (Daptacel), DTaP-IPV/Hib (Pentacel), pneumococcal (PCV13—Prevnar13), Td (Tenivac), Tdap (Adacel)
aluminium hydroxide	DTaP (Infanrix), DTaP-IPV (Kinrix), Hep A (Havrix), Hep B (Engerix-B), human papillomavirus (HPV) (Cervarix), Tdap (Boostrix)
aluminium potassium sulfate	DT, DTaP (Tripedia), Td (Decavac)
aluminium hydroxide, aluminium phosphate	DTaP-HepB-IPV (Pediarix), Hib (PedvaxHIB)
potassium aluminium sulfate, amorphous aluminium hydroxy phosphate-sulfate	Hib/Hep B (Comvax), Hep B (Recombivax)
amorphous aluminium hydroxy phosphate-sulfate	Hep A (Vaqta), HPV (Gardasil)
aluminium phosphate + aluminium hydroxide	Hep A/Hep B (Twinrix)

*Gallium* (*Z*=31) compounds are used in therapy and in diagnosis [314–316]. The radionuclide ^68^Ga (half-life 1.1 h, decays by electron capture) can be efficiently generated using a ^68^Ge/^68^Ga generator, removing the need for radiopharmacies to have a cyclotron on site [317]. Its short half-life makes ^68^Ga-labelled radiopharmaceuticals popular for clinical use, e.g. tumour imaging [314,318]. ^67^Ga (half-life 3.3 days, electron capture followed by γ emission) scintigraphy is used in oncology to detect malignant tumours in patients [314,319], including Hodgkin's and non-Hodgkin's lymphomas [320].

Gallium nitrate (Ga(NO_3_)_3_, Ganite) can suppress the growth of subcutaneously implanted tumours [314]. It has completed a phase I clinical trial for treatment of children with brain tumours, neuroblastoma, rhabdomyosarcoma, non-Hodgkin's lymphoma and refractory solid tumours [319] and phase II trials in patients with relapsed or refractory non-Hodgkin's lymphoma [321,322]. During studies of the antineoplastic activity of GaNO_3_, it was noted that calcium levels in blood decreased in many patients [323], prompting investigations into the management of elevated blood calcium levels associated with cancer [324]. Gallium nitrate is also currently in phase I clinical trials for patients suffering from cystic fibrosis [325]. Non-redox-active Ga^3+^ can inhibit (redox-active) Fe(III)-dependent pathways, including iron pathways in bacteria [326].

*Indium* (*Z*=49) has no known biological role in humans. The radionuclide ^111^In, a γ-emitter with a half-life of 2.8 days, is used in clinical diagnostic imaging. For example, ^111^In-CHX-A DTPA Trastuzumab (Indium-Herceptin) is about to enter clinical trials for imaging breast cancer [327] and a phase II trial is evaluating the side effects of administration of ^111^In ibritumomab tiuxetan in combination with ^90^Y ibritumomab tiuxetan, rituximab, fludarabine, melphalan for stem cell transplants in patients with B-cell non-Hodgkin lymphoma [328].

*Thallium* (*Z*=81) is best known for its toxicity, especially as Tl^+^ when it can interfere with K^+^ pathways (sometimes called ‘super-potassium’). The isotope ^205^Tl (70% abundant, nuclear spin 1/2) is sensitive for NMR detection. ^201^Tl (half-life 73 h) which decays by electron capture is used in radiodiagnostic imaging (SPECT).

## 6. Group 14: the carbon family

*Carbon* (*Z*=6) makes up 18.5% of the body and is the second most abundant element in humans, where its oxidation states range from +4 (CO_2_, bicarbonate) to −4 (hydrocarbons). We will not discuss the ‘organic’ chemistry of life and medicines here which is, of course, extensive, but just mention recent biological and medical interest in carbon monoxide.

The body produces about 3–6 ml of CO per day from the breakdown of haem by the enzyme haem oxygenase. Since carbon monoxide is a natural metabolite with signalling functions in the body, it is of interest as a drug [329]. In particular, metal carbonyl complexes such as [Ru(CO)_3_Cl(glycinate)] (CORM-3) hold potential as carbon monoxide releasing molecules (CORMs) for the transport and delivery of CO to target sites. Such drug use may be protective against transplant organ rejection, inflammation, and endothelial oxidative damage, and perhaps provide antibiotics to combat infection [330,331].

*Silicon* (*Z*=14) is present at significant levels in humans (total *ca* 21 g), but it is unclear if this element is essential to our function or not. No specific genes coding for Si are currently known for humans. Some fibrous silicate asbestos minerals are known to be health hazards [332].

Over the last three decades, much research has gone into ascertaining the importance of Si in our bodies. Silicon may be essential for bone formation [333], for synthesis of collagen and its stabilization, and matrix mineralization [333]. The ingestion of bioavailable silicon may increase bone mineral density, and Si supplementation in both animals and humans has a direct positive effect on bone density and strength [334].

**Figure 20.**
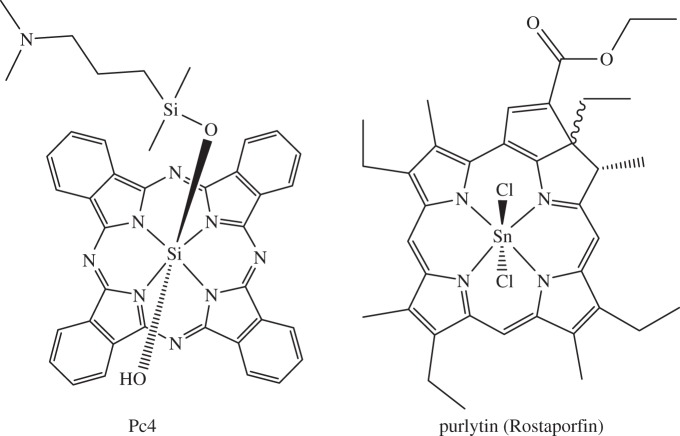
Structures of the photosensitizers Pc4 and purlytin.

A silicon-containing phthalocyanine (Pc4, figure 20) is a second generation photosensitizer currently in clinical trials, and can kill tumour cells and lymphoid cells via apoptosis [335,336]. Pc4 has completed phase I clinical trials for photodynamic therapy of patients with actinic keratosis, Bowen's disease, skin cancer, or stage I or stage II mycosis fungoides [337–339] and is entering phase I clinical trials for photodynamic therapy in stage IA–IIA cutaneous T-cell non-Hodgkin lymphoma [340].

There is no evidence to support the essentiality of *germanium* (*Z*=32) for man; however, germanium-containing preparations are sold as health elixirs and with claimed benefits for several illnesses including cancer and AIDS [341,342]. Cases of renal failure and even death, nephropathy, anaemia, myopathy and gastrointestinal disturbances have been reported after germanium intoxication [342,343], although the mutagenicity, carcinogenicity and teratogenicity of germanium compounds appear to be generally low. [344]. Bis-2-carboxyethylgermanium sesquioxide (Ge-132) is of current interest in relation to the treatment of cancer, burns, hepatitis and some cardiovascular diseases [345,346].

Forty years ago, *tin* (*Z*=50) was thought to be essential for the growth of animals and predicted to be essential for humans, but now there is little evidence that this is the case. We are widely exposed to tin through dinnerware and the cans used food packaging, and there are trace amounts of tin in the body (*ca* 16 mg). The only current use of Sn in clinical medicine is in the photodynamic therapy agent purlytin (Rostaporfin, Sn^IV^ ethyl etiopurpurin). Purlytin (figure 20) underwent clinical trials for different cancers, curtaneous basal cell cancer, kapososis sarcomas in AIDS patients and for breast metasteses in the chest wall [347]. Many organotin compounds have anti-cancer and antiviral activity, but none has reached clinical trials [348–352]. Recent examples include tri-^n^butyltin(IV)lupinylsulfide hydrogen fumarate which is active against leukaemia and melanoma *in vitro* and *in vivo*.

In general, *lead* (*Z*=82) is very toxic to humans. If inhaled or ingested, it can affect the nervous system, and long-term exposure can result in a plethora of adverse effects including anaemia, nephropathy, colic-like abdominal pains and brain damage [353–355]. In adults, almost 99% of absorbed lead accumulates in erythrocytes for between 30 and 35 days allowing it to be dispersed to soft tissues such as the liver, aorta, brain, lung and spleen [356]. Organo-lead compounds such as alkyl-Pb^IV^ compounds are metabolized to neurotoxic metabolites. Formation of these metabolites is catalysed in the liver by cytochrome p450-dependent monooxygenases [356]. Lead inhibits the steps in haem synthesis catalysed by ALA dehydratase and ferrochelatase.

The radioisotope ^212^Pb (half-life 10.6 h, β^−^ emission, rapidly generating the *α*-emitter ^212^Bi) is of interest for targeted *α*-particle therapy and radioimmunotherapy [357–359]. The decay leads to the emission of two short-lived α-particles that have strong therapeutic activity on cellular nuclei [357]. The radiolabelled compound ^212^Pb-TCMC-trastuzumab shows promising pre-clinical anti-tumour activity, binding to the extracellular domain of human epidermal growth factor receptor 2 (HER2). HER2 is a tyrosine kinase receptor that is overexpressed on the cell surface of many different cancer cell types, and upon internalization ^212^Pb then delivers a dose of α-radiation [360].

## 7. Group 15: the nitrogen family (pnictogens)

The human body is *ca* 3% (total *ca* 2 kg) *nitrogen* (*Z*=7), present in many biomolecules including amino acids, proteins, nucleobases, RNA and DNA, making it an essential element. A wide range of oxidation states are important in N biochemistry ranging from −3 (NH_3_) to +5 (nitrate).

**Scheme 1.**
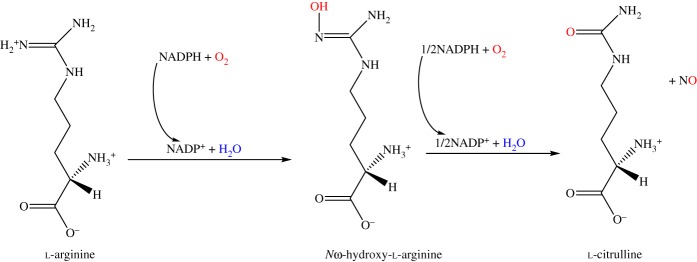
Production of NO by NOS enzymes [362].

The inorganic chemistry of nitric oxide (NO) has attracted recent attention. This reactive free radical is an important signalling molecule in the body, responsible for regulation of numerous biological processes including neurotransmission, smooth muscle contraction and immune reactions [361]. It is produced via conversion of l-arginine to l-citrulline by nitric oxide synthases (NOS) using NADPH and oxygen (scheme 1) [362]. Three NOS isoforms have been identified, endothelial (eNOS), neuronal (nNOS) and inducible (iNOS) [363]. Neuronal NOS is responsible for producing NO in nervous tissue and is involved in cell communication, eNOS generates NO in blood vessels and participates in vasodilation, and iNOS is located in the cardiovascular and immune systems and upon stimulation by proinflammatory cytokines produces large quantities of NO [363].

^13^N is a positron emitting isotope used in PET imaging. It has an extremely short half-life of 10 min and is therefore generated onsite in cyclotrons. ^13^N-ammonia has been in use since the 1970s for cardiac PET imaging [364]. It is administered by injection and exists as an equilibrium mixture of ^13^NH_3_ (minor) and 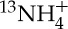 (major) in the bloodstream [365]. The neutral ^13^NH_3_ molecule can easily diffuse across plasma and cell membranes and once inside myocytes re-equilibrates with its protonated form which is then trapped in glutamine via the enzyme glutamine synthase [365]. Toxic ammonia is converted into urea which is excreted by the kidneys, via the urea cycle involving six enzymes and two mitochondrial transporters.

There is *ca* 0.9 kg of the essential element *phosphorus* (*Z*=15) in the body, up to 90% of which is in apatite in bones and teeth and the rest in extracellular fluids and soft tissue [366]. Phosphorus(III) has a well-established chemistry but is too strong a reductant to be found in most living organisms. Phosphorus(V) plays a major role in biological molecules, particularly in phosphate diester linkages in the structural framework of DNA and RNA. Polyphosphate esters are used by living cells for transport of cellular energy, especially ATP. Disruption of phosphorus homeostasis in the body can have detrimental consequences. Hypophosphatemia, low levels of phosphate in serum, has been suggested to lead to rhabdomyolysis, respiratory failure, haemolysis and cardiac failure [367]. Hyperphosphatemia (increased levels of phosphate in serum) has been linked to chronic kidney disease [368].

Bone formation from cross-linked collagen, osteocalcin and osteopontin proteins together with an equal amount of calcium phosphate mineral is carefully controlled in osteoblast cells. Osteoblast cells break down bone as part of the continual dynamic remodelling and repair processes. Phosphorus-32 (half-life 14.3 days) is a β-emitting radionuclide used in therapeutic and diagnostic oncology as well as general biochemical tracing. It is used for detection of malignant tumours, as cancer cells accumulate more phosphate than normal cells [369]. Phosphocol P32 is a ^32^P radiolabelled drug that is used to treat different cancers and as a therapeutic for cancer-related effects such as fluid build-up in the pleura or peritoneum (http://www.mayoclinic.org/drugs-supplements/chromic-phosphate-p-32-injection-route/description/drg-20062817 (accessed 26 August 2014)).

*Arsenic* (*Z*=33) is probably most widely known for its poisonous effect in humans. Symptoms of acute arsenic poisoning include diarrhoea, vomiting, blood in the urine, cramping muscles and eventually, death. Arsenic is an ultra-trace element in the body and normal blood levels in humans are 4–30 nM with a half-life of *ca* 2 h [370]. Chronic arsenic exposure is a serious problem in many parts of the world [371]. When elevated levels of As are consumed, it is enzymatically converted to monomethyl- and dimethyl-arsenic species by arsenite methyltransferase (encoded by the As3MT gene), primarily in liver cells [372] and rapidly excreted in the urine. Arsenate decreases ATP levels by unravelling its synthesis through a biochemical phenomenon known as arsenolysis and produces As^III^. Arsenic(III) has a high affinity for thiols and can inhibit enzymes such as pyruvate dehydrogenase [373].

**Figure 21.**
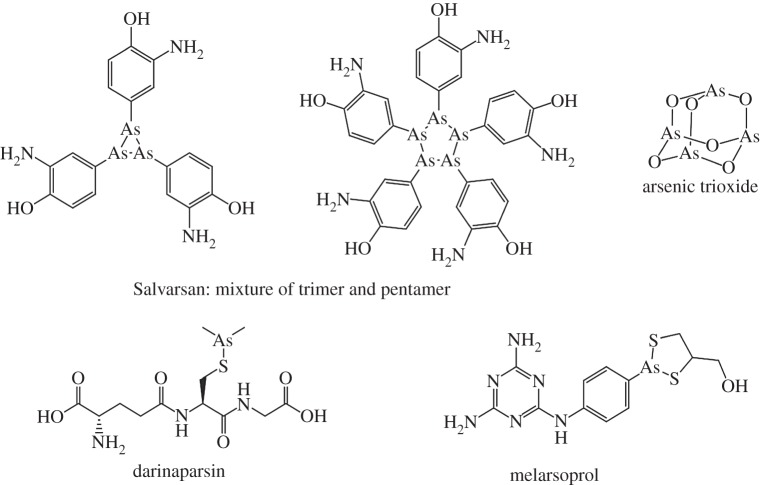
Structures of arsenic-containing drugs [370].

Despite its toxic reputation, arsenic compounds do have some therapeutic efficacy and were employed as far back as the eighteenth century when a potassium bicarbonate-based solution of arsenic trioxide (As_2_O_3_) (figure 21) known as Fowler's solution was prescribed for a variety of illnesses [374]. Salvarsan and Neosalvarsan were used in the early twentieth century as antibiotics to treat syphilis [375]. Arsenic trioxide (As_2_O_3_) is an approved drug for management of promyelocytic leukaemia [376,377] and is in clinical trials for treatment of several other cancers including unresectable hepatocellular carcinoma and non-small-cell lung cancer [378,379]. In water, As_2_O_3_ forms As(OH)_3_ which can enter cells via glycerol transport pathways (aquaglyceroporins). Melarsoprol is an organoarsenical that is used to treat trypanosome infections in Africa [380]. Darinaparsin, *S*-dimethylarsino-glutathione, is undergoing clinical trials as a cancer therapeutic [381,382]. This drug is able to induce greater intracellular arsenic accumulation and cell death *in vitro* with lower systemic toxicity compared to arsenic trioxide [383].

**Figure 22.**
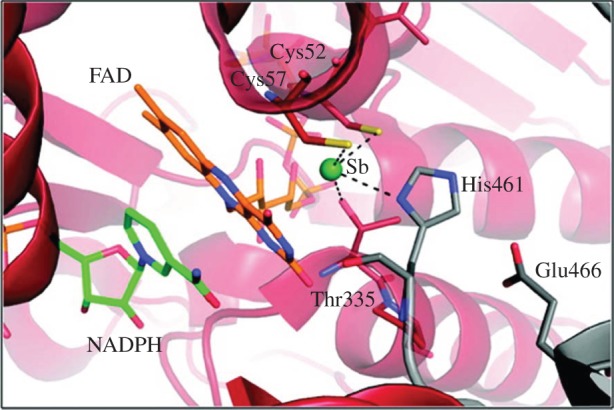
Reduced trypanothione reductase from *Leishmania infantum* bound to NADPH and Sb^III^. Sb^III^ is coordinated to the two redox-active catalytic cysteine residues (Cys52 and Cys57), Thr335 and His461 [389]. Reprinted with permission from Baiocco P, Colotti G, Franceschini S, Ilari A. 2009 *J. Med. Chem.* **52**, 2603–2612. Copyright • 2009 American Chemical Society.

*Antimony* (*Z*=51)-containing compounds have been used in medicine for centuries. The most significant application is the use of organoantimonials for the treatment of leishmaniases [384,385]. Antimony(III) potassium tartrate was prescribed in the early twentieth century for muco-cutaneous leishmaniasis and visceral leishmaniasis [384]. Unfortunately, there were severe side effects and less toxic Sb^V^ compounds were introduced in the 1940s [384]. Two formulations are still in use for leishmaniasis therapy, meglumine antimoniate (Glucantime) and sodium stibogluconate (Pentostam). It is proposed that Sb^V^ is a prodrug, reduced to Sb^III^ by the parasite or host cell [386]. The target of these antimonials is believed to be trypanothione, a low molecular weight thiol abundant in the *Leishmania* parasite [387]. Along with trypanothione reductase, trypanothione affords an intracellular reducing environment to circumvent oxidative stress thus ensure parasite survival [387,388]. Experimental studies point to Sb^V^ being reduced to Sb^III^ intracellularly followed by binding of Sb^III^ to trypanothione to form a complex that can then inhibit trypanothione reductase via thiolate exchange (figure 22) [388].

Prolonged exposure to antimony is a risk for people working in metal mining, smelting and refining, refuse incineration, coal-fired power plants and indoor firing ranges and may result in respiratory irritation, pneumoconiosis, antimony spots on the skin and gastrointestinal symptoms [390]. In a therapeutic setting, toxic side-effects of antimonials are cardiotoxicity and pancreatitis both of which are commonly observed in HIV and visceral leishmaniasis co-infections [390].

*Bismuth* (*Z*=83) compounds have long been used as antibacterial agents and are common today as a medication to eliminate *Helicobacter pyroli*, a Gram-negative bacterium that is the causative agent of gastrointestinal illnesses such as peptic ulcers [391]. Colloidal bismuth subcitrate (De-Nol) and bismuth subsalicylate (Pepto-Bismol) are used for prevention and treatement of gastric and duodenal ulcers [392,393]. Sulbogin is a topical ointment used for wound healing, and contains bismuth subgallate as one of its main components [394]. Bismuth subgallate acts through activation of the Hageman factor (factor XII) in the clotting cascade to quicken coagulation [395]. Bi^3+^ is a highly acidic metal ion (p*K*_a_ of aqua complex *ca* 1.5) and often forms polymeric complexes with hydroxide or oxide bridges, or citrate bridges in the case of bismuth subcitrate.

Interest in bismuth compounds as anti-cancer agents is growing due to the observation that administration of bismuth prior to chemotherapy can decrease the toxic side-effects associated with cisplatin [396,397]. Bismuth-213 is an *α*-emitter with a half-life of 45.6 min, prepared for clinical use from a ^225^Ac/^213^Bi generator. A phase I trial of ^213^Bi-lintuzumab demonstrated that administration of the radiopharmaceutical was safe up to 37 MBq kg^−1^ in patients with relapsed or refractory AML [398]. The phase I/II trial of sequential administration of cytarabine and ^213^Bi-lintuzumab for *α*-targeted radiotherapy in patients with AML found that ^213^Bi-lintuzumab gave rise to remissions in patients [399].

## 8. Group 16: the chalcogens

*Oxygen* (*Z*=8), the third most abundant in the universe (after H and He) and most abundant element in the Earth's crust by weight, is vital as O_2_ to support cell respiration. We breathe air that is 21% O_2_ by volume. Over half our body mass is oxygen (49/80 kg). Ground state O_2_ is a triplet (^3^O_2_) and relatively unreactive, even though a strong oxidant. Excited state singlet oxygen (^1^O_2_) in contrast is highly damaging and can kill cells (the basis of photodynamic therapy). The oxidation states of oxygen in the body range from 0 (O_2_) to −2 (H_2_O).

Oxygen homeostasis is crucial for survival of all vertebrates. Our bodies have evolved to ensure optimal oxygenation of all our cells, starting with entry through our lungs and then circulation and delivery of oxygen within the body [400]. Oxygen is a critical electron acceptor in redox reactions that lead to the production of ATP, the direct source of energy needed for cell function [401]. Oxygen is also an important substrate for manufacture or decomposition of many cellular components, including signalling mediators [401]. Hypoxia (lack of oxygen) is an important factor in development of severe pathologies including myocardial and cerebral ischaemia and cancer [400]. Hyperoxia (excess supply of O_2_ in tissues and organs) can also have detrimental consequences such as production of high concentrations of reactive oxygen species (ROS: ^1^O_2_, O_2_^−^, H_2_O_2_, OH^.^), disrupting the balance of oxidant and antioxidants and causing damage to cells and tissues [402].

Understanding the role of ROS species for signalling in the body is an important area of research [403]. Apart from its role as a destructive oxidant to ward off pathogens, H_2_O_2_ fulfils the role of ‘secondary messenger’: its enzymatic production and degradation as well as requirement for the oxidation of thiols give rise to the conditions appropriate for signalling [403]. The hypoxia-inducible factor (HIF) is a transcription factor that reacts to pathophysiologically low levels of oxygen via upregulation, setting off biological responses to hypoxia [404]. The HIF prolyl hydroxylase domain (PHD) enzymes selectively hydroxylate proline residues of HIF. The dependence of HIF protein levels on the concentration of O_2_ present, mediated by the PHD enzymes, forms the basis for one of the most significant biological sensor systems of tissue oxygenation in response to ischemic and inflammatory events [404]. As a result, pharmacological inhibition of these enzymes, resulting in stabilization of HIF, has therapeutic potential for treating conditions of tissue stress and injury [404].

*Sulfur* (*Z*=16) is an essential element, involved in numerous biochemical processes in oxidation states ranging from −2 (sulfide) to +6 (sulfate). It is the seventh most abundant element in the body (*ca* 160 g).

Sulfur compounds act as both electron donors and electron acceptors in metabolic reactions. Methionine (Met), the thioether amino acid, manages major metabolic and catalytic activities, initiates protein synthesis and undergoes reversible redox reactions to protect protein integrity [405]. The abundant intracellular thiol tripeptide glutathione (GSH) is a defensive biomolecule against toxic xenobiotics such as drugs, pollutants and carcinogens [406]. As an antioxidant, GSH participates in protection of the cell from oxidative stress either directly or as a cofactor of glutathione peroxidases [406]. Thioredoxins, with a dithiol-disulfide active site, are a class of redox proteins that participate in redox signalling in the body and are coded by the *TXN* gene [407]. Cysteine (Cys) is biosynthesized in humans and participates in enzymatic reactions as a nucleophile. Keratin's disulfide bonds are mostly responsible for the strength of this protein that is present in our skin, hair and nails.

Hydrogen sulfide (H_2_S), a highly toxic gas, is a mammalian messenger molecule. It is largely formed from Cys or its derivatives by the enzymes cystathionine β-synthase and cystathionine γ-lyase (CSE) [408]. One mechanism of H_2_S signalling is the sulfhydration (persulfide RSSH formation) of reactive Cys residues in target proteins [408]. This sulfhydration is responsible for the regulation of inflammation and endoplasmic reticulum stress signalling as well as of vascular tension [409]. A number of additional pharmacological targets for H_2_S have been identified including K(ATP), the ATP-sensitive potassium channel, and transient receptor potential channels, NF-κB nuclear translocation, extracellular signal-regulated kinase and Akt (protein kinase B) [410]. Atherosclerosis is a chronic disease that is considered to be the leading cause of cardiovascular morbidity and mortality [411]. Endogenous H_2_S production could be beneficial for prevention and treatment of atherosclerosis [412,413], since decreased levels of endogenous H_2_S induced by genetic deletion of CSE quickens the disease [412]. Sulfide is ubiquitous in the iron sulfur redox proteins ferredoxins. The Fe/S clusters are assembled on a scaffold protein in mitochondria, transported out and then incorporated into ferredoxins.

There is only about 4 mg of *selenium* (*Z*=34) in the body, but it is essential and its genetic codes are now well known. Selenium exerts its biological functions through selenoproteins.

Unlike the 20 standard amino acids, Sec is biosynthesized from serine on its tRNA. The insertion of Se donated by selenophosphate into the amino acid serine to give the 21st amino acid selenocysteine is encoded by the termination codon UGA, which is one of three termination codons for mRNA translation in non-selenoprotein genes, resulting in 25 selenoproteins with redox and signalling functions [414]. Recognition of the UGA codon as a Sec insertion site instead of stop requires a Sec insertion sequence element in selenoprotein mRNAs and a unique selenocysteyl-tRNA, both of which are recognized by specialized protein factors.

Selenium has an antioxidant effect as selenocysteine in the enzyme glutathione peroxidase (GSH-Px) and is also present in other selenoproteins such as the type 1 iodothyronine 59-deiodinase, important in the activation and deactivation of thyroid hormones [414]. However, the functions of most selenoproteins and Se-containing biomolecules still remain unclear. Selenomethionine and Se-methyl-seleno-l-cysteine are in phase I trials for chemopreventitive treatment of prostate cancer in healthy individuals [415]. Selenium sulfide is an antifungal agent that is used as an additive to shampoos to treat dandruff and certain types of dermatitis [416].

*Tellurium* (*Z*=52) has been regarded as a toxic, non-essential element but studies of the pharmacological properties of both organic and inorganic tellurium compounds have revealed some potential applications in medicine [417]. One such drug is AS101, a non-toxic Te^IV^ compound that is in phase II clinical trials for treatment of external genital warts, as an additive to the standard chemotherapy regimen of newly diagnosed elderly AML patients and AML transformed myelodysplastic syndrome patients and as a preventative agent of bone marrow toxicity due to chemotherapy in cancer patients [418–420]. A study of the oral and intraperitoneal administration of AS101 revealed that the drug significantly reduced clinical manifestations of inflammatory bowel diseases [421]. In a dextran sodium sulfate-induced colitis model, AS101 exerted its anti-inflammatory and anti-apoptotic activities through the downregulation of colonic cytokine levels (IL-17 and IL-1β) and by the blockade of leucocyte (neutrophil and macrophage) migration into the colon [421].

## 9. Group 17: the halogens

There is a lot of *fluorine* (*Z*=9) in the body (total *ca* 3 g) and in the form of fluoride may be essential, but surprisingly little appears to be known about specific uptake and transport mechanisms. There may be specific HF transport proteins (in acidic conditions, *vide infra*) and F^−^/H^+^ cotransporter or F^−^/OH^−^ antiporter proteins [422].

**Figure 23.**
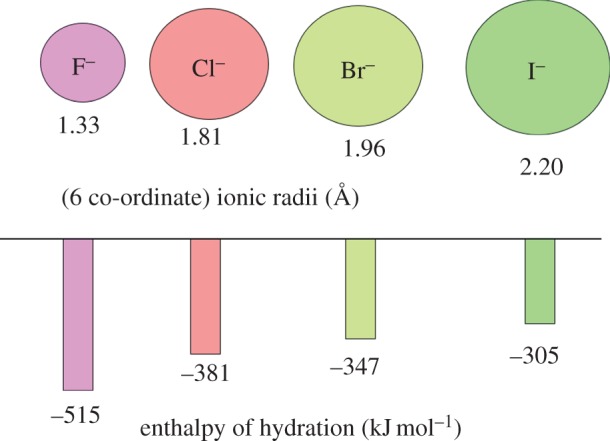
Ionic radii of the halide ions and enthalpy of hydration [27,28].

Fluoride is the smallest and most strongly basic of the halide ions, figure 23. Fluoride is added to drinking water and its presence has a beneficial effect on teeth and bones [423]. Fluoride has a high affinity for ‘hard’ metal ions such as Ca^2+^. It displaces hydroxyl ions in hydroxyapatite (Ca_5_(PO_4_)_3_OH), which is the main constituent of teeth and bones, to a stronger and harder fluoroapatite (Ca_5_(PO_4_)_3_F) [423]. It is recommended that small levels of 26–35 μM of fluoride can strengthen enamel. Gross systemic exposure to fluorides could alter bone homeostasis and dental enamel development [424]. Dental fluorosis is dependent on fluoride dose as well as timing and duration of exposure. In many parts of the world the amount of fluoride in ground water exceeds the recommended levels giving rise to fluorosis which then progresses to skeletal fluorosis.

Fluorides seem to regulate their activity through the mitogen-activated protein kinases MAPK signalling pathway which can result in alterations to gene expression, cell stress and cell death [424]. Fluoride metabolism in our bodies is pH dependent as the coefficient of permeability of lipid bilayer membranes to hydrogen fluoride is a million times that of the fluoride ion [425]. This translates to fluoride being able to readily cross cell membranes as HF in reaction to a pH gradient between body fluid compartments which is why following ingestion, levels of fluoride in blood plasma rapidly rise because of fast absorption in the stomach [425]. Aluminium fluoride and beryllium fluoride are used as inhibitors of many enzymes. They act as mimics of phosphate and can activate the heterotrimeric GTP-binding proteins [426]. There are more numerous fluorinated pharmaceuticals available as anaesthetics, antibiotics, anti-tumour and anti-inflammatories [427,428]. Fluorine-18 labelled deoxyglucose (electron capture, half-life 110 min) is one of the most widely used tracers for PET imaging to detect changes in glucose metabolism in pathophysiological processes [429]. ^18^F-fluorodeoxyglucose-positron emission tomography (^18^F-FDG-PET) is also used for screening, localization and follow-up of hypermetabolic processes including malignancies, infections and autoimmune processes [430].

*Chlorine* (*Z*=17) is prevalent in the body (*ca* 96 g). The concentration of Cl^−^ is high in body fluids (*ca* 100 mM in blood) and is regulated in cellular compartments (e.g. 25 mM in the cytoplasm and 4 mM in the nucleus of cells). Unlike fluorine, but in common with bromine and iodine, chlorine can exist in oxidized forms in the body.

Membrane transport proteins for Cl^−^ have been characterized. Epithelial transport of Cl^−^ (and SCN^−^) in many organs including the intestines, lungs, sweat glands and kidneys is mediated by the cystic fibrosis transmembrane conductance regulator (CFTR) [431], a 1480 amino acid cAMP-activated ATP-gated anion-channel glycoprotein. Mutations in the gene encoding CFTR can lead to reduction in either its transport capacity for chloride or cell surface expression causing the condition cystic fibrosis [432,433] and a type of male sterility as a result of congenital absence of the vas deferens [434].

Higher oxidation states of chlorine also play a role in the body. Hypochlorous acid (HOCl) is a strong oxidant (bleach!) produced by white blood cells (neutrophils) and can eradicate all types of bacteria. Even though it is employed by our immune system, it can also be lethal to our own cells depending on the dose [435]. In neutrophils, the haem enzyme myeloperoxidase uses hydrogen peroxide to convert chloride to HOCl [436].

There are significant amounts of *bromine* (*Z*=35) in the body (*ca* 0.2 g) and in blood (3.2–5.6 μg ml^−1^), arising from a daily dietary intake of bromide *ca* 2–8 mg (fish, grains and nuts), and there is growing evidence that it is essential. Eosinophil peroxidase is a haloperoxide enzyme encoded for by the *EPX* gene and produces both HOBr and HOCl [437]. Even though it can produce HOCl, it preferentially chooses bromide over chloride to produce brominating agents even at physiological concentrations, when chloride is vastly more abundant [438].

A recent paper has concluded that bromine is an essential trace element for all animals [439], since Br^−^ (on conversion to HOBr) is a required cofactor for peroxidasin-catalysed formation of sulfilimine cross-links in a posttranslational modification essential for tissue development within the collagen IV scaffold of basement membranes [439]. More research is needed to confirm the nutritional benefits of bromine.

**Figure 24.**
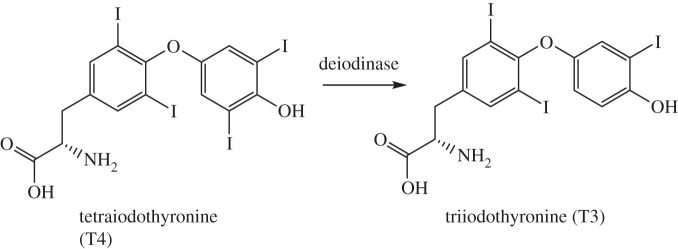
The three and four iodine atoms in triiodothyronine and thyroxine, respectively. T_3_ is the more active form of the hormone produced from T_4_ by the action of a Se-dependent deiodinase (peroxidase) enzyme.

Although there is only about 16 mg in the body, *iodine* (*Z*=53) is essential for the thyroid gland in the form of thyroid hormones, especially triiodothyronine (T3) (figure 24) and thyroxine (tetraiodothyronine, T4), synthesized from iodine (iodide) and tyrosine from the protein thyroglobulin by the enzyme thyroid peroxidase. These hormones play a major role in regulating growth and development. Iodine deficiency results in goitre (Derbyshire neck), avoided by supplementation of, e.g. table salt with iodate. The importance of C–I bonds can be contrasted with the behaviour of the lighter halogens in the body.

The Na^+^/I^−^ symporter (NIS) plays an important role in iodine metabolism and thryroid regulation [440]. NIS is an integral plasma membrane glycoprotein and thyroidal I^−^ transport from the bloodstream is made possible by selective targeting of NIS to the basolateral membrane [440]. It also regulates transport of I^−^ in other tissues, including salivary glands, gastric mucosa and lactating mammary gland, where I^−^ is translocated into the milk for thyroid hormone biosynthesis by the nursing newborn [441]. NIS is an ideal foundation for diagnostic and therapeutic management of thyroid cancer and its metastases using radioiodide [442]. Transduction of NIS into various kinds of cancer cells to render them susceptible to destruction with radioiodide has been reported [440,443]. Remarkably, more than 80% of human breast cancer samples express endogenous NIS, making the potential use of radioiodide for diagnosis and treatment possible [444].

*Astatine*-211 is an *α*-emitter with a half-life of 7.2 h. α-Emitters have unique features that make them ideal for elimination of focal pockets of tumour cells that are predominantly located in normal neutral tissue in the CNS [445]. For example, they can be used for targeted therapy without risk of adjacent normal tissue toxicity that sometimes occurs with β-emitters; this feature is ideal for sensitive areas such the CNS [445]. A pilot clinical study has evaluated the feasibility, safety and efficacy of the chimeric antitenascin mAb 81 C6 labelled with ^211^At in patients with recurrent malignant brain tumours [445]. The radiotherapeutic agent ^211^At-MX35 F(ab′)_2_ has also completed phase I pharmacokinetic studies in women in complete clinical remission of recurrent ovarian cancer [446].

## 10. Group 18: the noble gases

Exploration of the noble gases in medicine has been limited, although *helium* (*Z*=2) is being considered for adjunct therapy for respiratory ailments such as asthma, croup and bronchiolitis [447]. It has also shown efficacy in protection of the myocardium from ischaemia, though the mechanism of protection is unclear [411]. Employment of He in the operating theatre as a replacement for CO_2_ to insufflate the abdomens of patients undergoing laparoscopic abdominal procedures is being evaluated [448]. Helium is a better alternative as it prevents respiratory acidosis in patients with co-morbid conditions that cause CO_2_ retention [448]. Helium ion microscopy offers imaging of organs in very fine detail and hyperpolarized helium (HP ^3^He) for pulmonary MRI can be used for imaging of both normal and diseased lungs [447,449].

About 1% of the air we breathe is *argon* (*Z*=18), mostly arising from the radioactive decay of ^40^K in the Earth's crust. Argon has found application in electrosurgery; gas discharges can induce mainly superficial thermal effects on tissue in a non-contact manner (argon plasma coagulation) [450]. It was first used in open surgery in the 1970s and then later adapted for endoscopy [451,452]. Examples of its use include repair of haemorrhages that can occur during surgery or ulcers, injured blood vessels, varices and tumours, as well as devitalization and shrinkage of tumours and obstructing tissues [450].

*Krypton* (*Z*=36) gas, as the quadrupolar isotope ^83^Kr (nuclear spin *I*=9/2, natural abundance 11.5%) can be used for imaging (MRI) of airways particularly to differentiate between hydrophobic and hydrophilic surfaces [453,454].

*Xenon* (*Z*=54) is used as a general anaesthetic. It has a high affinity for the glycine site on the *N*-methyl-d-aspartate (NMDA) receptor, and unlike most other clinical NMDA receptor antagonists, it is not neurotoxic [455,456]. Of particular note is the use of xenon as an additive in the ventilation mix for a newborn baby to prevent brain injury (http://www.wales.nhs.uk/sitesplus/863/news/16104 (accessed 29 August 2014)). Xenon-133 (half-life 5.2 days, β^−^ decay) is used in radioimaging of the lungs by SPECT [457], and measurement of blood flow [458]. Hyperpolarized ^129^Xe (nuclear spin *I*=1/2, 26% abundant) is an MRI contrast agent used for imaging of the flow of gas in the lungs [459].

*Radon* (*Z*=86) is a toxic radioactive (dense) gas produced from the breakdown of long-lived (billions of years) radioactive uranium (and thorium) via ^226^Ra in soils, rocks and water. Decay of radon yields radioactive solids (radon daughters) which are *α*-emitters and attach to dust particles and are then inhaled. This inhalation has been linked to lung cancer [460,461]. Radon exposure may also lead to production of toxic reactive oxygen species. Dissolved radon in a pregnant woman's bloodstream can have detrimental consequences including retardation of brain development and even death [460]. Accumulation of radon in homes is a potential problem. Prevalent isotopes are ^222^Rn (from ^238^U), an α-emitter with a half-life of 3.8 days, and ^220^Rn (α-emitter, half-life 56 s) from the *α*-decay of ^224^Ra.

## 11. Conclusion

We have reviewed very briefly the role of the elements of the periodic table in human life. In particular we have tried to identify which elements are essential for man and to indicate other elements which are or can be used in diagnosis and therapy. There is much medicinal inorganic chemistry still to be explored. The field is in its infancy compared to medicinal organic chemistry. More detailed background to the biological chemistry of the elements, including the influence of the environment on the natural selection of the elements by animals can be found in the recent publications by Williams *et al*. [462–464]. It is not easy to compile a definitive list of essential elements. Elements which are added to mineral supplements these days are largely there as a result of nutritional experiments carried out many years ago [5–20]. Such experimental studies on animal growth under defined nutritional conditions are expensive and the interdependence of the biochemistry of the elements and various organic cofactors can complicate the interpretation. Moreover, not only do we wish to know which elements are essential for normal growth, but also what their biochemical functions are and which particular species (chemical forms of the element) are essential.

There are many interesting examples of where a biological function requires the synergistic action of two or more elements. For example, a Se enzyme deiodinates the thyroid hormone T_4_ [465], copper ceruloplasmin oxidizes iron and assists with its transport [466], cytochrome oxidase has both copper and iron in its active site for conversion of oxygen into water, cytoplasmic superoxide dismutase uses both copper and zinc to convert superoxide into oxygen and water, and the close cooperation between iron (Fe^II^ and Fe^III^) and sulfur (sulfide and cysteine thiolate) is evident in ubiquitous ferredoxin proteins which are critical for electron storage and transport.

Since the sequence of the human genome (and many others) is now known, it is timely to ask the question ‘can we recognize genetic codes for essential elements?’ Our conclusions are listed in table 2, a list which produces 20 essential elements and six other potentially essential elements.

**Table 2.** Examples of human genetic (proteomic) codes for the elements of the periodic table.

atomic number	element	examples of genetic codes
1	H	uptake and transport of H-containing molecules (C−H, N−H, etc.), proteins, bicarbonate (CO_2_) for pH control
6	C	general: C−H, C=O, etc. taken up in building blocks (amino acids, fatty acids, etc.)
		regulation of CO_2_, carbonate, CO
7	N	general: N−H, N−C, etc., amino acids, nucleotides
		NOSs; conversion of NH_3_ into urea (enzymes and transporters)
8	O	for example, O_2_ uptake, transport (haemoglobin) and storage (myoglobin), conversion of O_2_ to H_2_O_2_, and H_2_O by mitochondrial proteins, various enzymes (e.g. superoxide dismutase, catalase)
		O_2_-sensor proteins (e.g. HIF)
9	F	F^−^/H^+^ cotransporter or a F^−^/OH^−^ antiporter ?? Biochemistry poorly understood
11	Na	membrane pumps Na^+^/K^+^ ATPase
12	Mg	MAGT1, magnesium transporter 1
15	P	kinases, phosphatases, nucleotides
16	S	thiol/disulfide and Met in proteins; sulfide in ferredoxins
17	Cl	chloride channel: CFTR
19	K	membrane pumps Na^+^/K^+^ ATPase
20	Ca	Ca^2+^-sensor protein troponin; Ca^2+^-ATPase membrane pump; calmodulin transduces Ca^2+^ signals in cells
25	Mn	most abundant Mn protein glutamine synthetase; Mn superoxide dismutase in mitochondria
26	Fe	non-haem Fe proteins *ca* 1% of human proteome
27	Co	uptake and carrier proteins for vitamin B12
29	Cu	Cu proteins *ca* 1% of human proteome
30	Zn	Zn^2+^ proteins *ca* 10% of human proteome
34	Se	25 selenoproteins with redox and signalling functions
42	Mo	four Mo enzymes (xanthine oxidoreductase and sulfite oxidase families); taken up only as molybdate?
53	I	thyroid hormones thyroxine (T4) and triiodothyronine (T3) synthesised from tyrosine residues in protein thyroglobulin
potentially essential elements		
14	Si	role in bone mineralization?
23	V	role in phosphate biochemistry?
24	Cr	influence on glucose metabolism?
28	Ni	essential for some microorganisms
		allergenic: MHCII-Ni-peptide recognition by T cells
35	Br	essential (as HOBr?) for killing invading microorganisms?
		essential for assembly of collagen IV scaffolds in tissue development—therefore essential for all animals?
50	Sn	once thought to be essential for animal growth

This short journey through the periodic table illustrates that, as yet, we are unable to understand the role of many elements found in the body in terms of genetic codes. Much modern drug design now relies on target-based design—drugs which target specific gene products (proteins/enzymes). The inventive use of minerals in medicines is therefore in danger of being abandoned. It is worth recalling the ancient Chinese approach—the use of *stone drugs* (*mineral drugs*) developed over a period of some 2000 years, preparations from natural sources (minerals and animal fossils) that in some cases contain up to 35 elements [467]. Synergistic effects may well arise from combinations of elements (and organic compounds such as vitamins). Many exciting challenges remain in this field.

## Footnotes

1 In this article, we have used the data on the abundances of elements in humans given in WebElements: http://www.webelements.com/ (for a body weight of 80 kg).
